# Sub-50 nm perovskite-type tantalum-based oxynitride single crystals with enhanced photoactivity for water splitting

**DOI:** 10.1038/s41467-023-43838-3

**Published:** 2023-12-05

**Authors:** Jiadong Xiao, Mamiko Nakabayashi, Takashi Hisatomi, Junie Jhon M. Vequizo, Wenpeng Li, Kaihong Chen, Xiaoping Tao, Akira Yamakata, Naoya Shibata, Tsuyoshi Takata, Yasunobu Inoue, Kazunari Domen

**Affiliations:** 1 Research Initiative for Supra-Materials, Interdisciplinary Cluster for Cutting Edge Research, Shinshu University, Nagano-shi, Nagano 380-8553 Japan; 2 Institute of Engineering Innovation, School of Engineering, The University of Tokyo, 2-11-16, Yayoi, Bunkyo-ku, Tokyo, 113-8656 Japan; 3 Graduate School of Natural Science and Technology, Okayama University, 3-1-1 Tsushimanaka, Kita-ku, Okayama 700-8530 Japan; 4 Japan Technological Research Association of Artificial Photosynthetic Chemical Process (ARPChem), 2-11-16 Yayoi, Bunkyo-ku, Tokyo, 113-8656 Japan; 5 Office of University Professors, The University of Tokyo, 2-11-16 Yayoi, Bunkyo-ku, Tokyo, 113-8656 Japan

**Keywords:** Photocatalysis, Nanoparticles

## Abstract

A long-standing trade-off exists between improving crystallinity and minimizing particle size in the synthesis of perovskite-type transition-metal oxynitride photocatalysts via the thermal nitridation of commonly used metal oxide and carbonate precursors. Here, we overcome this limitation to fabricate *A*TaO_2_N (*A* = Sr, Ca, Ba) single nanocrystals with particle sizes of several tens of nanometers, excellent crystallinity and tunable long-wavelength response via thermal nitridation of mixtures of tantalum disulfide, metal hydroxides (*A*(OH)_2_), and molten-salt fluxes (e.g., SrCl_2_) as precursors. The SrTaO_2_N nanocrystals modified with a tailored Ir–Pt alloy@Cr_2_O_3_ cocatalyst evolved H_2_ around two orders of magnitude more efficiently than the previously reported SrTaO_2_N photocatalysts, with a record solar-to-hydrogen energy conversion efficiency of 0.15% for SrTaO_2_N in Z-scheme water splitting. Our findings enable the synthesis of perovskite-type transition-metal oxynitride nanocrystals by thermal nitridation and pave the way for manufacturing advanced long-wavelength-responsive particulate photocatalysts for efficient solar energy conversion.

## Abstract (web summary)

A trade-off exists between improving crystallinity and minimizing particle size in the synthesis of perovskite-type transition-metal oxynitrides. The authors break this limitation to fabricate sub-50 nm ATaO2N (A = Sr, Ca, Ba) single nanocrystals exhibiting improved photocatalytic water-splitting performance

## Introduction

Perovskite-type oxynitrides of early transition metals and alkaline-earth or rare-earth elements, in optimal cases, can combine the advantages of oxides and nitrides, exhibiting greater stability in air and moisture than pure nitrides and narrower bandgaps than comparable oxides^1,2^. In recent decades, this superiority has made these perovskite-type compounds an important class of functional materials used in nontoxic pigments^3^, colossal magnetoresistors^4^, high-permittivity dielectrics^5^, and long-wavelength-responsive photocatalysts^6^. In response to an urgent worldwide need to reduce carbon emissions to mitigate global warming, perovskite oxynitride semiconductor-based particulate photocatalysts have attracted particular attention because they potentially enable the direct synthesis of sustainable fuels and chemical products using sunlight as the sole energy source^6–9^. The tantalum oxynitride perovskites, *A*TaO_2_N (*A* = Ca, Sr, Ba), are rare semiconducting materials with excellent stability in aqueous solutions, narrow bandgaps (1.9‒2.4 eV), and conduction and valence bands appropriately positioned to straddle the water redox potential^6,10^. As such, they are regarded as among the most promising photocatalyst materials for overall water splitting (OWS), which can be used in particulate-photocatalyst-sheet-based panel systems for large-scale solar H_2_ production^6,11^.

Nitridation of oxide (with carbonate) precursors in flowing ammonia (NH_3_) at high temperatures is the most widely applied method for the synthesis of perovskite oxynitrides^2^. Such oxynitrides, including *A*TaO_2_N (*A* = Sr, Ca, Ba) produced by thermal nitridation of polymetallic oxides, are generally polycrystalline and incorporate structural defects that act as recombination and trapping centers for photogenerated charge carriers^12,13^. This disadvantage can be overcome by using a mixture of polymetallic oxides (or metal oxides and carbonates) together with a molten-salt flux (e.g., NaCl, KCl, or RbCl) as the nitridation precursor^14–17^. The best-in-class *A*TaO_2_N semiconductors are prepared by this approach and are characterized by well-crystallized single crystals and improved photocatalytic activity^15–17^. However, the sizes of the resultant particles are uncontrollable and inevitably large (several hundred nanometers at minimum)^15–17^, leading to long distances for the photoexcited charge carriers to migrate to reach active sites on the surface. This problem points to a long-standing trade-off between improving crystallinity and minimizing particle size in the synthesis of metal oxynitride perovskites via the flux-assisted thermal nitridation process. Overcoming this limitation would represent a critical advancement in long-wavelength-responsive perovskite oxynitride-based photocatalyst manufacturing yet remains a grand challenge.

Here, we show that using a mixture of tantalum(IV) sulfide (TaS_2_), metal hydroxide (*A*(OH)_2_, A = Ca, Sr, Ba), and a molten salt (e.g., SrCl_2_) as the nitridation precursor enables the fabrication of highly crystalline *A*TaO_2_N (*A* = Ca, Sr, Ba) single nanocrystals with sub-50 nm particle sizes and a tunable long-wavelength response. Each precursor material was found to be critical to the formation of single nanocrystals that simultaneously exhibit a high degree of crystallinity and a small particle size of a few tens of nanometers. This approach leads to high photocatalytic efficiency of the resultant SrTaO_2_N nanocrystals toward sacrificial H_2_ and O_2_ evolution and Z-scheme water splitting. In particular, Ir–Pt alloy@Cr_2_O_3_-modified SrTaO_2_N nanocrystals evolved H_2_ approximately two orders of magnitude more efficiently than the previously reported SrTaO_2_N photocatalysts and were used, for the first time, as a H_2_-evolution photocatalyst (HEP) for Z-scheme water splitting, providing a solar-to-hydrogen (STH) energy conversion efficiency of 0.15%.

## Results and discussion

### Synthesis and characterization of *A*TaO_2_N (*A* = Sr, Ca, Ba) nanocrystals

**Fig. 1:**
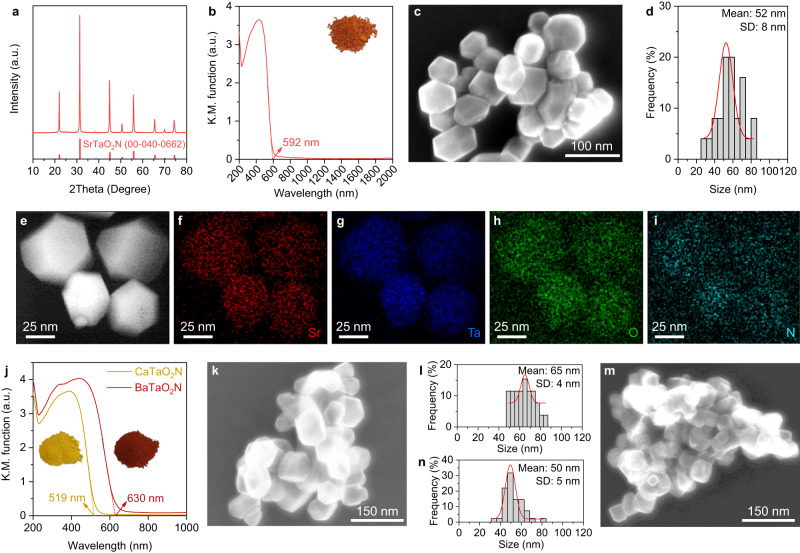
Synthesis and characterization of nanocrystalline *A*TaO_2_N (*A* = Sr, Ca, Ba). XRD pattern (**a**), UV–vis diffuse-reflectance spectrum (**b**), Scanning electron microscopy image (**c**), and particle size distribution (**d**) of the synthesized SrTaO_2_N. ADF–STEM image (**e**) and STEM–EDS elemental mapping images of Sr (**f**), Ta (**g**), O (**h**), and N (**i**) of a cross-sectional SrTaO_2_N sample. **j** UV–vis diffuse-reflectance spectra of the synthesized CaTaO_2_N and BaTaO_2_N. SEM image (**k**) and particle size distribution (**l**) of CaTaO_2_N. SEM image (**m**) and particle size distribution (**n**) of BaTaO_2_N. The mean value and standard deviation (SD) of the particle sizes in (**d**, **l**, **n**) were determined by Gaussian fitting (red lines). The insets in (**b**, **j**) show photographs of the respective materials.

Nitridation of a powder containing TaS_2_ (Supplementary Fig. 1), Sr(OH)_2_, and SrCl_2_ in a molar ratio of 1:2.5:1 under a flow of gaseous NH_3_ at 1223 K for 3 h yielded an orange powder (see details in Methods). The X-ray diffraction (XRD) pattern for the obtained powder indicated a single phase associated with perovskite-type SrTaO_2_N (Fig. 1a). The product also exhibited a light-absorption edge at approximately 600 nm, characteristic of SrTaO_2_N (Fig. 1b), and an average particle size of approximately 50 nm (Fig. 1c, d). Lattices fringes with the same orientation (Supplementary Fig. 2a) and a well-defined spot-like selected-area electron diffraction (SAED) pattern (Supplementary Fig. 2b) for a single particle, together with highly ordered lattice fringes at the outermost surface (Supplementary Fig. 3), were observed when a cross-sectional specimen of this material was examined by high-resolution transmission electron microscopy (HRTEM). On the basis of an elemental analysis, the chemical formula for the material was estimated to be Sr_1.00_Ta_1.01_O_2.07_N_1.03_ (Supplementary Table 1); the sulfur content (0.004 wt%) was below the detection limit (0.01 wt%). In keeping with this result, elemental mapping of a cross-sectional SrTaO_2_N sample using annular dark-field scanning transmission electron microscopy coupled with energy dispersive X-ray spectroscopy (ADF STEM–EDS) (Fig. 1e–i) indicated that Sr, Ta, O, and N were evenly distributed within single particles, whereas negligible sulfur was detected (Supplementary Fig. 4). Notably, the SrTaO_2_N nanocrystals exhibited extremely weak background absorption at longer wavelengths (>600 nm) (Fig. 1b), suggesting a low defect density^18,19^. These results indicate the formation of highly crystalline SrTaO_2_N single nanocrystals.

We found that each precursor component was critical to the formation of single nanocrystals of SrTaO_2_N with high crystallinity, and that the optimized TaS_2_/Sr(OH)_2_/SrCl_2_ molar ratio in the precursor was 1/2.5/1 (see details in Supplementary Figs. 5–7). A several-micron-sized 2H-TaS_2_ phase (Supplementary Fig. 1) has not been previously used for synthesizing perovskite-type oxynitrides; it was found to play an irreplaceable role in the developed approach. Nitridation of the Ta_2_O_5_/Sr(OH)_2_/SrCl_2_ (molar ratio: 0.5/2.5/1) mixture, where TaS_2_ was replaced with Ta_2_O_5_ (the most widely used Ta source for synthesizing oxynitrides^2,14,15^), did not produce SrTaO_2_N when reacted under the same conditions but generated predominantly Sr_6_Ta_2_O_10.188_ (Supplementary Fig. 5). Replacement of Sr(OH)_2_ with SrCO_3_ resulted in SrTaO_2_N nanoparticles but with relatively larger sizes (Supplementary Fig. 8). The molten-salt-assisted fragmentation of TaS_2_ under the nitridation conditions is presumably a key reason for the formation of SrTaO_2_N nanocrystals (see detailed discussion below Supplementary Fig. 9). The SrCl_2_ used as a molten-salt flux could be replaced with another congeneric flux. For instance, the use of NaCl, KCl, or RbCl instead of SrCl_2_ led to SrTaO_2_N nanocrystals with an average particle size of approximately 20 nm (Supplementary Figs. 10 and 11). However, the crystallinity of the resultant SrTaO_2_N samples was lower, as indicated by the large increase in the full-width at half-maximum (FWHM) of their characteristic XRD peaks, associated with a decrease in the melting point of the molten-salt flux (see explanation below Supplementary Fig. 10).

We readily extended the proposed approach to the synthesis of CaTaO_2_N and BaTaO_2_N nanocrystals by simply replacing Sr(OH)_2_ in the precursor with Ca(OH)_2_ and Ba(OH)_2_, respectively (Supplementary Fig. 12). The obtained yellow-colored CaTaO_2_N and crimson-colored BaTaO_2_N (Fig. 1j) exhibited average particle sizes of ~65 (Fig. 1k, l) and 50 nm (Fig. 1m, n), respectively. The light-absorption edges for the prepared CaTaO_2_N and BaTaO_2_N were located at 519 and 630 nm (Fig. 1j), respectively, whereas the typical values are 500 nm for CaTaO_2_N and 650 nm for BaTaO_2_N^6^. This difference is attributable to the slight substitution of Ca^2+^ and Ba^2+^ in the oxynitrides by Sr^2+^ as a result of the use of the molten-SrCl_2_ flux. Accordingly, the XRD peak positions for the CaTaO_2_N and BaTaO_2_N samples were shifted to lower and higher angles, respectively (Supplementary Fig. 12). These results demonstrate that nitridation of mixtures of TaS_2_, metal hydroxides, and molten-salt fluxes can be a universal and flexible approach for fabricating perovskite-type tantalum oxynitride nanocrystals with tunable light-absorption edge wavelengths.

### H_2_-evolution and Z-scheme water-splitting performance of the SrTaO_2_N-nanocrystal photocatalyst

**Fig. 2:**
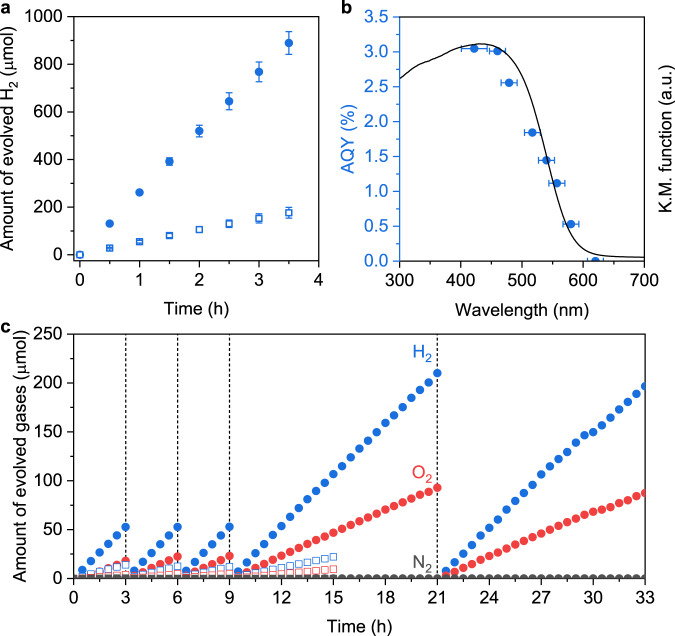
H_2_-evolution and Z-scheme OWS performance of SrTaO_2_N nanocrystal photocatalyst. **a** Time courses of the photocatalytic H_2_-evolution reaction in aqueous methanol (13 vol%) solution over the Cr_2_O_3_/Pt (MW_EG_)/Ir (MW_H2O_)-modified SrTaO_2_N nanocrystals (solid circles) and over the Cr_2_O_3_/Pt (MW_EG_)/Ir (MW_H2O_)-modified large-sized SrTaO_2_N crystals (Supplementary Fig. 13) prepared from the Ta_2_O_5_/SrCO_3_/SrCl_2_ precursor (open squares). Error bars indicate the standard deviation of three measurements. **b** AQY as a function of the incident-light wavelength during visible-light-driven H_2_ production over Cr_2_O_3_/Pt (MW_EG_)/Ir (MW_H2O_)-modified SrTaO_2_N nanocrystals. The solid line indicates the UV–vis diffuse-reflectance spectrum of SrTaO_2_N. The above reactions were carried out under illumination from a Xe lamp (300 W, *λ* ≥ 420 nm) with or without various bandpass filters. **c** Time courses of gas evolution during photocatalytic Z-scheme OWS under simulated sunlight using Cr_2_O_3_/Pt (MW_EG_)/Ir (MW_H2O_)-modified SrTaO_2_N nanocrystals (solid circles) or large-sized SrTaO_2_N (precursor: Ta_2_O_5_/SrCO_3_/SrCl_2_, Supplementary Fig. 13) (open squares) as the HEP, Ir-CoFeO_*x*_/BiVO_4_ as the OEP, and [Fe(CN)_6_]^3−^/[Fe(CN)_6_]^4−^ as a redox mediator. Conditions: HEP, 50 mg; Ir-CoFeO_*x*_/BiVO_4_, 100 mg; 150 mL of 25 mM phosphate buffer solution (pH = 6) containing K_4_[Fe(CN)_6_] (5 mM); light source, solar simulator (AM 1.5 G, 0.87 sun); irradiation area for solar simulator, 9.3 cm^2^; background pressure, 5 kPa.

As a demonstration, the H_2_-evolution activity of SrTaO_2_N nanocrystals was evaluated after the nanocrystals were sequentially modified with Ir and Pt by microwave heating in water and ethylene glycol (EG), respectively, and finally with Cr_2_O_3_ by photodeposition (see details in Methods). The obtained photocatalyst (denoted as Cr_2_O_3_/Pt (MW_EG_)/Ir (MW_H2O_)/SrTaO_2_N nanocrystals) was found to evolve H_2_ efficiently from an aqueous methanol solution (solid circles in Fig. 2a) in which the feeding concentrations of Ir, Pt, and Cr (relative to the mass of SrTaO_2_N) were optimized to be 0.5, 1.0, and 0.5 wt%, respectively (Supplementary Fig. 13). The H_2_-evolution rate for the Cr_2_O_3_/Pt (MW_EG_)/Ir (MW_H2O_)/SrTaO_2_N nanocrystals was approximately five times higher than that for the SrTaO_2_N modified with the same cocatalysts but synthesized by nitridation of a typical Ta_2_O_5_/SrCO_3_/SrCl_2_ precursor and exhibiting an average particle size of 200 nm (open squares in Fig. 2a; see characterization results in Supplementary Fig. 14). Moreover, the single-nanocrystal SrTaO_2_N evolved H_2_ four times higher than the polycrystalline SrTaO_2_N exhibiting aggregates composed of several polycrystalline nanoparticles with an average size of 63 nm (see detailed discussion in Supplementary Fig. 15) and 2.4 times higher than the SrTaO_2_N previously developed from a Ta_2_O_5_/NaOH/SrCl_2_ precursor^17^ (Supplementary Fig. 16). These results demonstrate the importance of both a small particle size and a high degree of crystallinity to the photocatalytic performance and the superiority of the developed approach in producing highly crystalline single nanocrystals of *A*TaO_2_N (*A* = Sr, Ca, Ba). The onset irradiation wavelength for H_2_ generation agreed with the absorption edge for this SrTaO_2_N photocatalyst (Fig. 2b), indicating that the photoreaction proceeded via bandgap transitions. The associated apparent quantum yield (AQY) for H_2_ evolution was calculated to be 3.0% at 422 nm, 2.6% at 479 nm, and 0.5% at 580 nm (Fig. 2b). Notably, the previously reported SrTaO_2_N photocatalysts evolve H_2_ inefficiently with a rate well below 5 µmol h^−1^, and the associated AQY is too low to be detected (Supplementary Table 2). An estimation based on the evolution rates for H_2_ indicates that the developed Cr_2_O_3_/Pt (MW_EG_)/Ir (MW_H2O_)/SrTaO_2_N nanocrystal photocatalyst evolves H_2_ approximately two orders of magnitude more efficiently than the previously reported SrTaO_2_N photocatalysts (Supplementary Table 2).

When the H_2_-evolving Cr_2_O_3_/Pt (MW_EG_)/Ir (MW_H2O_)/SrTaO_2_N nanocrystals were combined with the reported Ir and FeCoO_*x*_ nanocomposite co-modified BiVO_4_ (Ir-FeCoO_*x*_/BiVO_4_)^20^ (see characterization results in Supplementary Fig. 17) as the O_2_-evolution photocatalyst (OEP) and [Fe(CN)_6_]^3−^/[Fe(CN)_6_]^4−^ as a redox mediator, both H_2_ and O_2_ were stably evolved under simulated sunlight at a near-stoichiometric molar ratio of 2:1 for 33 h with negligible deactivation (Fig. 2c). Upon modification with the same Cr_2_O_3_/Pt (MW_EG_)/Ir (MW_H2O_) cocatalyst, the SrTaO_2_N nanocrystals (solid circles in Fig. 2c) exhibited notably higher photocatalytic activity in Z-scheme water splitting than the large-sized SrTaO_2_N prepared from the typical Ta_2_O_5_/SrCO_3_/SrCl_2_ precursor (open squares in Fig. 2c). The STH energy conversion efficiency for this redox-mediated Z-scheme system was 0.15%, and the AQY was measured to be 4.0% at approximately 420 nm (Supplementary Fig. 18). These STH and AQY values are still lower than those for Z-scheme systems constructed with Rh_*y*_Cr_2−*y*_O_3_-loaded ZrO_2_-modified TaON (AQY of 12.3% at 420 nm and STH efficiency of 0.6%)^20^ or Ru-modified SrTiO_3_:La,Rh (AQY of 33% at 419 nm and STH of 1.1%)^21^ as HEPs. Nevertheless, this AQY is comparable to that for the Z-scheme system constructed with Pt/BaTaO_2_N as the HEP^15^, both representing the most efficient bias-free Z-scheme water-splitting systems involving 600-nm-class photocatalysts. More importantly, our proposed Z-scheme system is, to the best of our knowledge, the first involving SrTaO_2_N as the HEP because previous SrTaO_2_N photocatalysts evolved H_2_ inefficiently (Supplementary Table 2). Further improvements are expected by refining the operating parameters and exploring long-wavelength-responsive OEPs and more effective redox mediators or solid conductive mediators.

### Nanostructure and promotion effects of Cr_2_O_3_/Pt (MW_EG_)/Ir (MW_H2O_) cocatalyst

**Fig. 3:**
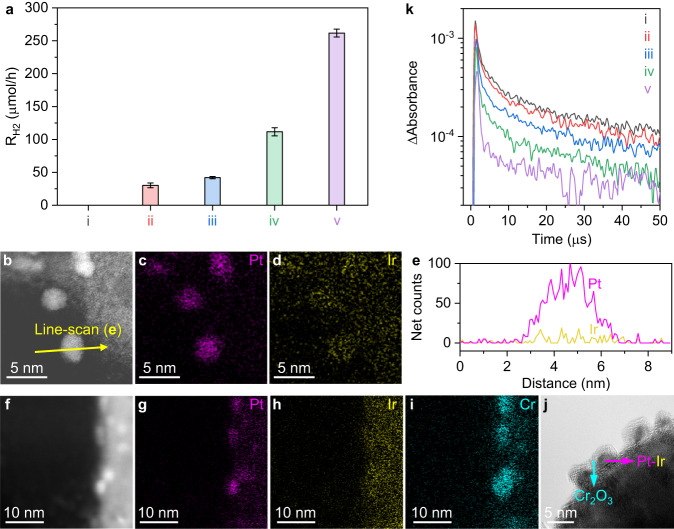
Nanostructure and characteristics of Cr_2_O_3_/Pt (MW_EG_)/Ir (MW_H2O_) cocatalyst promoting H_2_ evolution. **a** Photocatalytic H_2_-evolution rate (calculated in the first one hour) for bare and different cocatalyst-modified SrTaO_2_N nanocrystals: i, bare SrTaO_2_N; ii, Pt (MW_EG_)/SrTaO_2_N; iii, Pt (MW_EG_)/Ir (MW_H2O_)/SrTaO_2_N; iv, Cr_2_O_3_/Pt (MW_EG_)/SrTaO_2_N; v, Cr_2_O_3_/Pt (MW_EG_)/Ir (MW_H2O_)/SrTaO_2_N. Error bars indicate the standard deviation of three measurements. ADF STEM image (**b**), and Pt (**c**) and Ir (**d**) EDS mapping images of a cross-sectional Pt (MW_EG_)/Ir (MW_H2O_)/SrTaO_2_N sample. **e** Pt and Ir EDS line-scan spectra across the nanoparticle marked with a yellow arrow in (**b**). ADF STEM image (**f**), and Pt (**g**), Ir (**h**), and Cr (**i**) EDS mapping images of a cross-sectional Cr_2_O_3_/Pt (MW_EG_)/Ir (MW_H2O_)/SrTaO_2_N sample. **j** HRTEM image of the surface of a Cr_2_O_3_/Pt (MW_EG_)/Ir (MW_H2O_)-modified SrTaO_2_N nanocrystal. **k** TA kinetic profiles of photoexcited electrons probed at 2000 nm over bare and different cocatalyst-modified SrTaO_2_N nanocrystals in subfigure (**a**).

The substantial improvement in the H_2_-evolution and Z-scheme water-splitting activity of the SrTaO_2_N nanocrystals also relied on the multicomponent Cr_2_O_3_/Pt (MW_EG_)/Ir (MW_H2O_) cocatalyst that can accelerate the surface H_2_-evolution reactions much more efficiently than Pt, a representative H_2_-evolution cocatalyst (Fig. 3a). Specifically, the addition of Ir and Cr_2_O_3_ increased the H_2_-evolution rate approximately twofold (photocatalyst vi versus v in Fig. 3a) and sixfold (photocatalyst iii versus v in Fig. 3a), respectively, compared with the case when either of them were absent. Because individual Ir- or Cr_2_O_3_-modified SrTaO_2_N exhibited negligible activities toward H_2_ evolution (Supplementary Fig. 19), the promotion effect of both components most likely resulted from their interactions with Pt that promote charge separation and transfer and/or promote surface reactions. This result motivated us, above all, to investigate the nanostructure of the Cr_2_O_3_/Pt (MW_EG_)/Ir (MW_H2O_) cocatalyst and, in particular, the interactions between different cocatalyst components.

Decoration with Ir (MW_H2O_) formed highly dispersed IrO_2_ species according to X-ray photoelectron spectroscopy (XPS) (Supplementary Fig. 20a) and ADF STEM (Supplementary Fig. 21a) analyses. Further decoration with Pt (MW_EG_) via a microwave heating process with EG as a reducing agent generated noticeable tiny nanoparticles on the surface of the SrTaO_2_N (Supplementary Fig. 21b). This step not only produced metallic Pt (Supplementary Fig. 22a) but also reduced approximately one-half of the previous IrO_2_ decoration species to metallic Ir (Supplementary Fig. 20c). This result is consistent with the observation that, when Ir (MW_H2O_)**/**SrTaO_2_N was subjected to a similar EG-mediated microwave treatment (denoted as Ir (MW_H2O_-MW_EG_)**/**SrTaO_2_N), approximately 43% of the IrO_2_ species was reduced to metallic Ir (Supplementary Fig. 20b). Interestingly, the XPS 4 *f* peak position for Ir^0^ in Pt (MW_EG_)/Ir (MW_H2O_)/SrTaO_2_N (Supplementary Fig. 20c) was negatively shifted by approximately 0.8 eV compared with that for Ir^0^ in the Ir (MW_H2O_-MW_EG_)**/**SrTaO_2_N specimen (Supplementary Fig. 20b). Meanwhile, the XPS 4 *f* peak position for Pt^0^ in Pt (MW_EG_)/Ir (MW_H2O_)/SrTaO_2_N (Supplementary Fig. 22b) was positively shifted by approximately 0.6 eV compared with that for Pt^0^ in the Pt (MW_EG_)**/**SrTaO_2_N specimen (Supplementary Fig. 22a). These shifts clearly point to strong electronic interactions between Ir^0^ and Pt^0^ species in Pt (MW_EG_)/Ir (MW_H2O_)/SrTaO_2_N, which indicates the formation of metal alloys^17,22,23^. STEM–EDS mapping and line-scan images (Fig. 3b–e) further indicate that the fed Ir and Pt species in Pt(MW_EG_)/Ir (MW_H2O_)/SrTaO_2_N dominantly form Ir–Pt alloy nanoparticles because both the Ir and Pt elements were confined across the area of individual nanoparticles. Cr_2_O_3_ was lastly photodeposited, forming Ir–Pt alloy@Cr_2_O_3_ core–shell nanostructured particles in Cr_2_O_3_/Pt (MW_EG_)/Ir (MW_H2O_)/SrTaO_2_N, as is evident in the STEM–EDS elemental maps (Fig. 3f–i) and HRTEM image (Fig. 3j). Notably, on the basis of the identification of both Cr_2_O_3_ and Cr(OH)_3_ species by XPS (Supplementary Fig. 23) and previous studies^6,24^, the Cr_2_O_3_ shell was composed of amorphous Cr(III)O_1.5−*m*_(OH)_2*m*_·*x*H_2_O and the Cr_2_O_3_ decoration did not alter the chemical state of Ir (Supplementary Fig. 20d) or Pt (Supplementary Fig. 22c).

The transient absorption (TA) kinetic profiles probed at 2000 nm (Fig. 3k) reflect the intraband transition of long-lived electrons in oxynitride materials^15,25^. The observed decrease in the TA signal intensity upon decoration with a cocatalyst indicates electron transfer from the SrTaO_2_N to the cocatalyst for the water reduction reaction. The slight decrease (from line i to ii in Fig. 3k) in the TA signal intensity upon decoration with Pt (MW_EG_) indicates that electrons were not effectively injected into the Pt. Decoration with Pt (MW_EG_)/Ir (MW_H2O_) resulted in a more substantial decrease (from line i to iii in Fig. 3k) in the TA signal intensity. This result confirms that the Ir–Pt alloy acts as a more efficient electron collector and H_2_-evolution catalyst than Pt alone, consistent with the higher H_2_-evolution rate for Pt(MW_EG_)/Ir (MW_H2O_)/SrTaO_2_N compared with that for Pt(MW_EG_)/SrTaO_2_N (Fig. 3a). The most notable decrease in the TA signal intensity was observed when Pt or Ir–Pt alloy was capped with a Cr_2_O_3_ overlayer (from line ii to iv and from iii to v in Fig. 3k). This result provides direct evidence that Cr_2_O_3_ greatly promotes electron transfer, consistent with our recent finding that a Cr_2_O_3_ coating on Rh nanoparticles on a fluorine-doped tin oxide (FTO) substrate substantially enhanced the current density^26^. The enhancement was most likely attributable to the Cr_2_O_3_ overlayer inhibiting hole transfer from the vicinity of the Pt or Ir–Pt alloy nanoparticles and thereby greatly reducing charge recombination at the metal nanoparticles or the SrTaO_2_N–metal interfaces. Moreover, the Cr_2_O_3_ shell is known to function as a molecular sieve to allow the permeation of H^+^, H_2_, and H_2_O species while preventing the oxidized species generated by holes from reaching the metal core and being reduced back^24,26,27^. Therefore, the deposition of Cr_2_O_3_ improved the H_2_-evolution rate approximately sixfold (Fig. 3a) and notably improved the photocatalytic durability by suppressing unfavorable side reactions (Supplementary Fig. 24).

### O_2_-evolution performance of CoO_*x*_-modified SrTaO_2_N nanocrystals

**Fig. 4:**
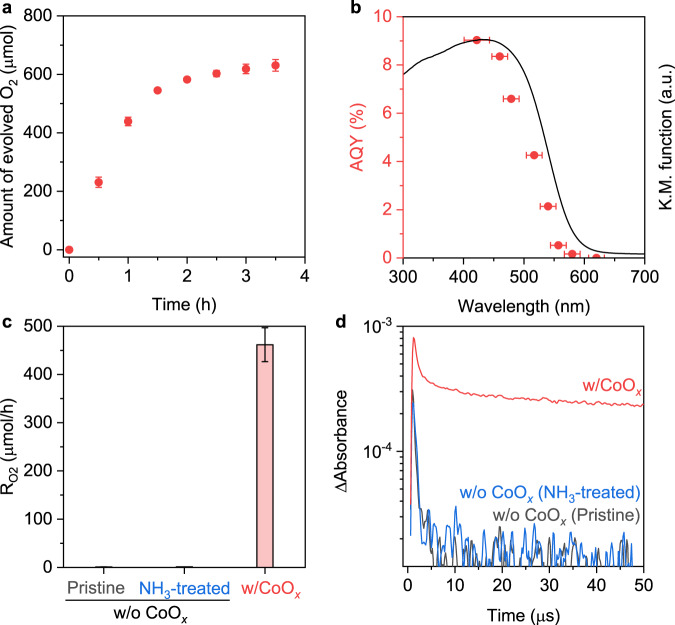
O_2_-evolution performance of SrTaO_2_N nanocrystal photocatalyst. Time course (**a**) of photocatalytic O_2_-evolution reaction in 20 mM AgNO_3_ solution over a CoO_*x*_-modified SrTaO_2_N photocatalyst under visible-light irradiation and the associated AQY at various wavelengths (**b**). The above reactions were carried out under illumination from a Xe lamp (300 W, *λ* ≥ 420 nm) with or without various bandpass filters. The solid line in (**b**) indicates the UV–vis diffuse-reflectance spectrum of SrTaO_2_N. Photocatalytic O_2_-evolution rates calculated at 0.5 h (**c**) and TA kinetic profiles of photoexcited electrons probed at 5000 nm (**d**) over pristine and NH_3_-treated SrTaO_2_N without CoO_*x*_ and over CoO_*x*_/SrTaO_2_N. Error bars in (**a**, **c**) indicate the standard deviation of three measurements.

Upon modification with a classic cobalt oxide (CoO_*x*_) cocatalyst (Supplementary Fig. 25) with an optimized feed concentration of 1.0 wt% (relative to the mass of SrTaO_2_N; Supplementary Fig. 26), the SrTaO_2_N nanocrystals also exhibited high photocatalytic O_2_-evolution performance from an aqueous AgNO_3_ solution (Fig. 4a). The associated AQYs were estimated to be 9.0%, 6.6%, and 0.2% at 422, 479, and 580 nm, respectively (Fig. 4b). These values are higher than most of those reported for SrTaO_2_N-based OEPs with CoO_*x*_ as the cocatalyst (Supplementary Table 2). This result demonstrates again the importance of the developed approach to afford high-quality SrTaO_2_N single nanocrystals. Consistent with the findings of previous studies^16^, CoO_*x*_ is critical for achieving efficient O_2_ evolution (Fig. 4c) because it can efficiently capture holes for the water oxidation reaction. Interestingly, the TA signal intensity probed at 5000 nm (Fig. 4d), which reflects the dynamics of free and/or shallowly trapped electrons^28^, was enhanced by approximately one order of magnitude following decoration by CoO_*x*_. This suggests that one-step-excitation OWS over this material will be a viable process in the near future if these populous long-lived electrons resulting from the CoO_*x*_ modification can be further extracted and used for H_2_ evolution via appropriate surface modification and/or cocatalyst design.

This work demonstrates that highly crystalline single nanocrystals of perovskite-type tantalum oxynitrides can be easily synthesized by thermal nitridation of mixtures of TaS_2_, metal hydroxides, and molten salts. Upon modification with a tailored Ir–Pt alloy@Cr_2_O_3_ cocatalyst, the SrTaO_2_N nanocrystals produced by this approach evolved H_2_ around two orders of magnitude more efficiently than the previously reported SrTaO_2_N photocatalysts, with an apparent quantum yield of 3% at the wavelength of 420 nm, from a methanol aqueous solution, and an STH energy conversion efficiency of 0.15% in Z-scheme water splitting. The CoO_*x*_-modified SrTaO_2_N nanocrystals also evolved oxygen efficiently, surpassing most of the reported SrTaO_2_N photocatalysts. We envision that appropriate surface and cocatalyst modifications would enable efficient one-step-excitation OWS over these *A*TaO_2_N (*A* = Sr, Ca, Ba) nanocrystals in the near future. The proposed synthetic strategy is expected to be applicable to a broad range of perovskite-type transition-metal oxynitride single nanocrystals, and can advance the manufacturing of long-wavelength-responsive particulate photocatalysts for efficient solar energy conversion.

## Methods

### Synthesis of nanocrystalline *A*TaO_2_N (*A* = Sr, Ca, Ba)

*A*TaO_2_N (*A* = Sr, Ca, Ba) nanocrystals were synthesized by heating of a mixture of TaS_2_, *A*(OH)_2_, and SrCl_2_ with a molar ratio of 1:2.5:1 under gaseous NH_3_ flow. In a typical synthesis of SrTaO_2_N nanocrystals, 1.20 g of TaS_2_ (99%; Kojundo Chemical Laboratory, Supplementary Fig. 1), 3.25 g of Sr(OH)_2_·8H_2_O (90.0+%; FUJIFILM Wako Pure Chemical), 0.78 g of SrCl_2_ (98.0%; Kanto Chemical), and 5 mL of ethanol were well mixed in an agate mortar with the aid of sonication and agitation. After desiccation by a mild heating process, the resultant powder was loaded into an alumina crucible and heated at 1223 K for 3 h under a 200 mL min^−1^ flow of gaseous NH_3_. SrTaO_2_N nanocrystals were obtained after the resultant solids were rinsed and then dried overnight at 313 K under vacuum. CaTaO_2_N and BaTaO_2_N nanocrystals were synthesized by a similar procedure in which Ca(OH)_2_ (99.9%; FUJIFILM Wako Pure Chemical) and Ba(OH)_2_·8H_2_O (98.0%; FUJIFILM Wako Pure Chemical) were used, respectively, instead of Sr(OH)_2_·8H_2_O.

### Cocatalyst modification for SrTaO_2_N

For H_2_-evolution reactions, SrTaO_2_N was decorated with Ir and Pt sequentially by microwave heating in water and EG, respectively, and finally with Cr_2_O_3_ by photodeposition via a modified version of a recently reported method^29^. The resultant material is denoted herein as Cr_2_O_3_/Pt (MW_EG_)/Ir (MW_H2O_)/SrTaO_2_N. In a typical process, SrTaO_2_N nanoparticles were first dispersed in distilled water (15 mL) containing the desired amount of IrCl_3_·3H_2_O (99.9%; Kanto Chemical) and the suspension was subsequently heated at 423 K for 10 min using a microwave reactor (Anton Paar, Monowave 200) while the suspension was stirred at 1000 rpm. After the sample cooled naturally, it was washed, filtered, and then dried at 313 K under vacuum to obtain Ir (MW_H2O_)/SrTaO_2_N. This sample was further subjected to similar microwave heating in a mixture of 13 mL of EG and 2 mL of distilled water containing the required amount of H_2_PtCl_6_·6H_2_O (>98.5%; Kanto Chemical) under the same conditions, resulting in Pt (MW_EG_)/Ir (MW_H2O_)/SrTaO_2_N. After washing and filtration, the wet Pt (MW_EG_)/Ir (MW_H2O_)/SrTaO_2_N was dispersed in an aqueous methanol solution (13 vol%) containing the required amount of K_2_CrO_4_ (99.0%; Kanto Chemical). After complete degassing, the suspension was irradiated with visible light (*λ* ≥ 420 nm) for 0.5 h; Cr_2_O_3_/Pt (MW_EG_)/Ir (MW_H2O_)-modified SrTaO_2_N was obtained.

For O_2_-evolution reactions, SrTaO_2_N was modified with the CoO_*x*_ cocatalyst by impregnation, followed by heating under a gaseous NH_3_ flow. A quantity of the SrTaO_2_N powder was immersed in an aqueous solution containing the required amount of Co(NO_3_)_2_·6H_2_O ( > 98.0%; Kanto Chemical) as a Co precursor, and the resultant slurry was continuously stirred with intense sonication for 2 min to disperse the SrTaO_2_N. After the slurry was dried on a hot-water bath, the powder sample was heated at 1173 K for 1 h under a 200 mL min^−1^ flow of gaseous NH_3_ to obtain the CoO_*x*_/SrTaO_2_N nanoparticulate photocatalyst.

### Preparation of Ir-CoFeO_*x*_/BiVO_4_

Ir-CoFeO_*x*_/BiVO_4_ was prepared according to the method reported elsewhere^20^. NH_4_VO_3_ (10 mmol; 99.0%; FUJIFILM Wako Pure Chemical) and Bi(NO_3_)_3_·5H_2_O (10 mmol; 99.5%; FUJIFILM Wako Pure Chemical) were dissolved in 2.0 M nitric acid solution, whose pH was then adjusted to ~0.5 by addition of an NH_3_ solution (25–28 wt%). The mixed solution was strongly stirred until a light-yellow precipitate was observed; the precipitate was further aged for ~2 h and then transferred to a Teflon-lined stainless-steel autoclave for a 24 h hydrothermal treatment at 473 K. BiVO_4_ was obtained after the collected powder was washed with distilled water and dried in vacuum at 313 K for 6 h. The BiVO_4_ was further suspended in 150 mL of 25 mM phosphate buffer solution (pH = 6) containing a calculated amount of Na_2_IrCl_6_ (0.8 wt% Ir, relative to the mass of BiVO_4_), Co(NO_3_)_2_ (0.2 wt% Co, relative to the mass of BiVO_4_), and K_3_[Fe(CN)_6_] (0.1 mM). After irradiation with UV–vis light for 2 h under static air conditions, Ir-CoFeO_*x*_/BiVO_4_ was collected, washed, and dried for further use.

### Characterization of materials

The crystal phases were characterized by XRD analysis using a Rigaku MiniFlex 300 powder diffractometer equipped with a Cu Kα radiation source (*λ* = 1.5418 Å). Diffuse-reflectance spectra were acquired with an ultraviolet–visible–near-infrared spectrometer (V-670, JASCO) and converted from reflectance into the Kubelka–Munk function. The contents of Sr and Ta metals in the SrTaO_2_N were determined by inductively coupled plasma atomic emission spectroscopy (ICP-AES; ICPS-8100, Shimadzu). The oxygen, and nitrogen contents of the SrTaO_2_N were determined with an oxygen–nitrogen analyzer (Horiba, EMGA-920); while the sulfur content was determined with a carbon-sulfur analyzer (Horiba, EMIA-Pro). XPS analysis was carried out using a PHI Quantera II spectrometer equipped with an Al Kα radiation source. All binding energies were referenced to the C 1 *s* peak (284.8 eV) arising from adventitious carbon. Scanning electron microscopy (SEM) and TEM images were acquired with a Hitachi HD-2300A scanning transmission electron microscope using the SEM and TEM modes, respectively. STEM images, EDS mapping images and SAED patterns were recorded using a JEOL JEM-ARM200F Cold FE (Cs-STEM), a JEOL JEM-ARM200F Thermal FE (Cs-STEM) and a JEOL JEM-2800 equipped with an Oxford Instruments X-MAX 100TLE SDD detector, respectively. The cross-sectional sample for STEM observation was made by Ar ion milling using a JEOL EM-09100IS ion slicer.

Mid-infrared (IR) time-resolved TA spectroscopic investigations were performed using a pump–probe nanosecond system equipped with a Nd:YAG laser (Continuum, Surelite I; duration: 6 ns) and custom-built spectrometers^17,28,30^. Photoexcited charge carriers were monitored in the mid-IR region, with probe energies from 6000 to 1200 cm^−1^ (from 1667 to 8333 nm), providing dynamics of photogenerated electrons in the photocatalysts^17,28,30^. The IR probe light from a MoSi_2_ coil was focused on the photoexcited sample, and the transmitted IR beam was then passed through the monochromatic grating spectrometer. The transmitted light was subsequently detected by a mercury-cadmium-telluride detector (Kolmar). To excite the photocarriers in SrTaO_2_N with and without cocatalysts, 440 nm laser pulses with a fluence of 500 μJ pulse^−1^ generated from an optical parametric oscillator were used. The output electrical signal was amplified using an alternating-current coupled amplifier (Stanford Research Systems (SR560), bandwidth: 1 MHz). The time resolution of the spectrometer was limited to 1 μs by the bandwidth of the amplifier. For data acquisition, 1000 TA signals were accumulated to generate a decay profile at the probe wavelength. For preparation of the sample film, a suitable amount of bare or cocatalyst-loaded SrTaO_2_N was dispersed on water and then drop-cast onto a CaF_2_ substrate to obtain a film with a density of 1.09 mg cm^−2^; the film was then dried in air overnight. TA spectroscopic measurements were carried out under N_2_ ambient (20 Torr) and at room temperature.

### Photocatalytic reactions of H_2_ and O_2_ evolution and Z-scheme OWS

All photocatalytic reactions were carried out at 288 K under a background pressure of 5 kPa in a Pyrex top-illuminated reaction vessel connected to a closed gas circulation system. For the photocatalytic H_2_-evolution reaction, 150 mg of photocatalyst was well dispersed in 150 mL of an aqueous methanol solution (13 vol%) without adjustment of the pH. After air was completely removed from the reaction slurry by evacuation, Ar gas was introduced to generate a background pressure of approximately 5 kPa and the reactant solution was irradiated with a 300 W Xe lamp equipped with a cold mirror and a cut-off filter (L42, *λ* ≥ 420 nm). For the photocatalytic O_2_-evolution reaction, 150 mg of photocatalyst together with 100 mg of La_2_O_3_ as a pH buffer was well dispersed in 150 mL of an aqueous 20 mM AgNO_3_ solution. After air was completely removed from the reaction vessel, the photocatalyst suspension was irradiated with a 300 W Xe lamp equipped with a cold mirror and a cut-off filter (L42, *λ* ≥ 420 nm). For the photocatalytic Z-scheme OWS reaction, Cr_2_O_3_/Pt (MW_EG_)/Ir (MW_H2O_)-modified SrTaO_2_N (50 mg) as the HEP and Ir-FeCoO_*x*_-modified BiVO_4_ (100 mg) as the OEP were dispersed in 150 mL of 25 mM sodium phosphate buffer solution (pH = 6.0) containing K_4_[Fe(CN)_6_] (5 mM). After air was completely removed from the reaction slurry by evacuation, Ar gas was introduced to generate a background pressure of approximately 5 kPa and the suspension was irradiated by a solar simulator (SAN-EI Electronic, XES40S1, AM 1.5 G, 87 mW cm^−2^). The top window of the reaction vessel was covered with a mask to confine the irradiated sample area to 9.3 cm^2^. The gaseous products evolved during these reactions were analyzed using an integrated online gas chromatography system consisting of a GC-8A chromatograph (Shimadzu) equipped with molecular sieve 5 Å columns and a thermal conductivity detector, with Ar as the carrier gas.

### AQY measurements

The AQY for the photocatalytic reactions was calculated according to the equation

$${{{{{\rm{AQY}}}}}}(\%)=[A\times R]/I\times 100,$$

where *R* and *I* are the rate of gas evolution and the incident photon flux, respectively, and *A* is the number of electrons needed to generate one molecule of H_2_ or O_2_ (i.e., two or four for photocatalytic sacrificial H_2_ or O_2_ evolution, respectively, and four for H_2_ evolution in Z-scheme water splitting based on two-step photoexcitation). The photocatalytic reactions were carried out using the same experimental setup and conditions described above, except for the use of bandpass filters with central wavelengths of 422, 460, 479, 517, 540, 557, 580, and 620 nm. The FWHM of the 422 nm bandpass filter was 21 nm, whereas that of the other bandpass filters was 13 nm. The number of incident photons was measured using an LS-100 grating spectroradiometer (EKO Instruments).

### STH energy conversion efficiency measurements

The water-splitting reaction was performed under simulated solar radiation generated with a solar simulator (SAN-EI Electronic, XES40S1, AM 1.5 G). The STH conversion efficiency was calculated as

$${{{{{\rm{STH}}}}}}(\%)=({R}_{{{{{{\rm{H}}}}}}2}\times \varDelta G)/(P\times S)\times 100,$$

where *R*_H2_, Δ*G*, *P*, and *S* denote the rate of H_2_ evolution during the OWS reaction, the Gibbs energy for the OWS reaction (237 kJ mol^−1^ at 288 K), the energy intensity (87 mW cm^−2^) of the AM 1.5 G solar radiation used (equivalent to 0.87 sun), and the irradiated sample area (9.32 cm^2^), respectively.

### Supplementary information

Supplementary Information

Peer review file

### Source data

Source Data

## Data Availability

The authors declare that the data supporting the findings of this study are available within the paper and its Supplementary Information files. Source data are provided with this paper.
