# Supplementary material for: Sub-50 nm perovskite-type tantalum-based oxynitride single crystals with enhanced photoactivity for water splitting
Source: Nat Commun. 2023 Dec 5;14:8030. doi: 10.1038/s41467-023-43838-3 (PMC10696056; doi:10.1038/s41467-023-43838-3)
Supplement: Supplementary file 1 — Supplementary Information [file 41467_2023_43838_MOESM1_ESM.pdf]

Supplementary Information for

Sub-50 nm Perovskite-Type Tantalum-Based Oxynitride Single Crystals with Enhanced Photoactivity for Water Splitting

Jiadong Xiao[1], Mamiko Nakabayashi[2], Takashi Hisatomi[1], Junie Jhon M. Vequizo[1], Wenpeng Li[1], Kaihong Chen[1], Xiaoping Tao[1], Akira Yamakata[3], Naoya Shibata[2], Tsuyoshi Takata[1], Yasunobu Inoue[4], Kazunari Domen[1,5]*

[1] Research Initiative for Supra-Materials, Interdisciplinary Cluster for Cutting Edge Research, Shinshu University, Nagano-shi, Nagano 380-8553, Japan.

[2] Institute of Engineering Innovation, School of Engineering, The University of Tokyo, 2-11-16, Yayoi, Bunkyo-ku, Tokyo 113-8656, Japan.

[3] Graduate School of Natural Science and Technology, Okayama University, 3-1-1 Tsushimanaka, Kita-ku, Okayama 700-8530, Japan.

[4] Japan Technological Research Association of Artificial Photosynthetic Chemical Process (ARPChem), 2-11-16 Yayoi, Bunkyo-ku, Tokyo 113-8656, Japan.

[5] Office of University Professors, The University of Tokyo, 2-11-16 Yayoi, Bunkyo-ku, Tokyo 113-8656, Japan.

*Corresponding author.

Email: domen@shinshu-u.ac.jp

TABLE OF CONTENTS

Supplementary Figs. 1–26 Pages S1-S30
Supplementary Tables. 1–2 Pages S31-S32
Supplementary References Page S33

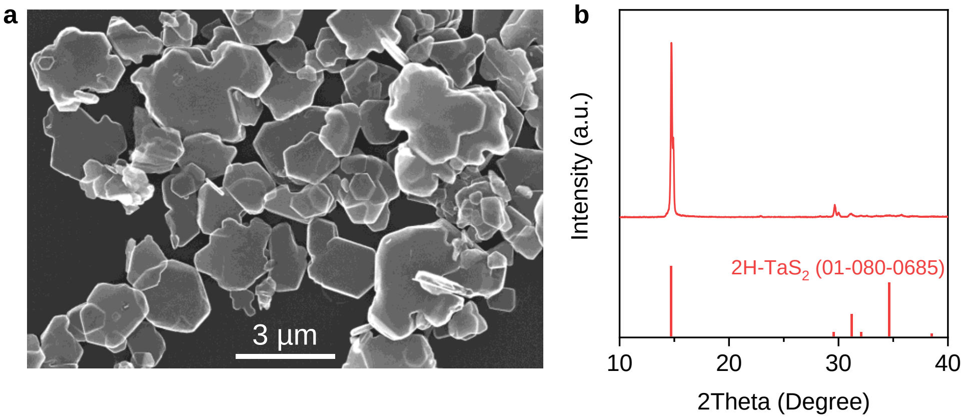

Supplementary Fig. 1. (a) SEM image and (b) XRD pattern for TaS_2 used in this work.

Note: The TaS_2 provided by Kojundo Chemical Laboratory exhibits a several-micron-sized stacked-layer structure and is composed predominantly of the 2H-TaS_2 phase.

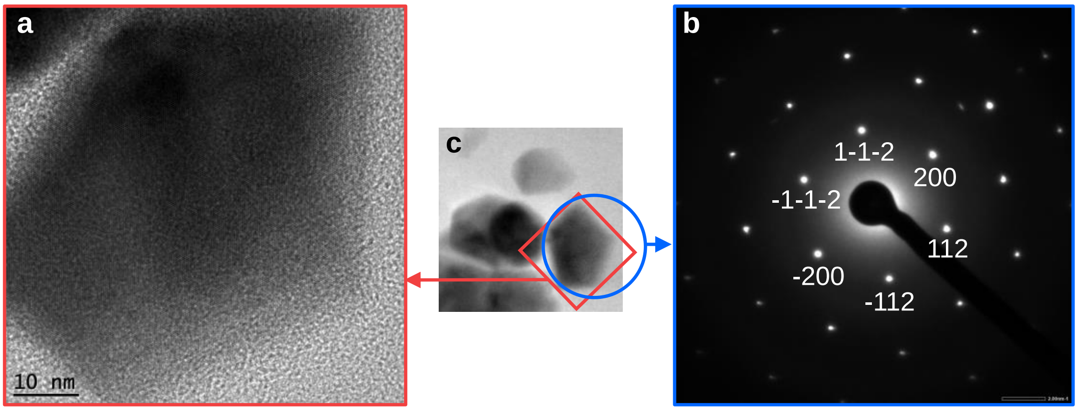

Supplementary Fig. 2. (a) HRTEM image and (b) SAED pattern for (c) a cross-sectional $SrTaO_2N$ particle.

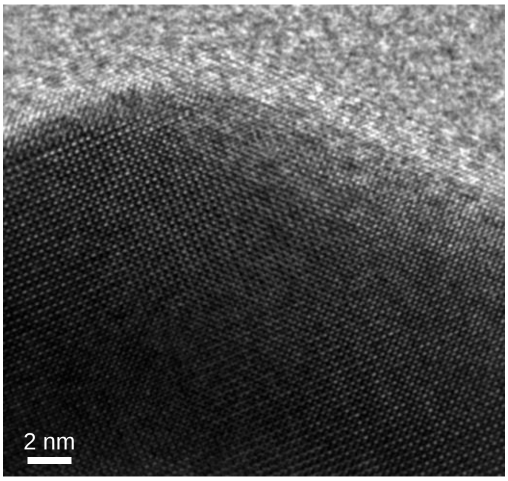

Supplementary Fig. 3. HRTEM image of the edge of a cross-sectional $SrTaO_2N$ particle.

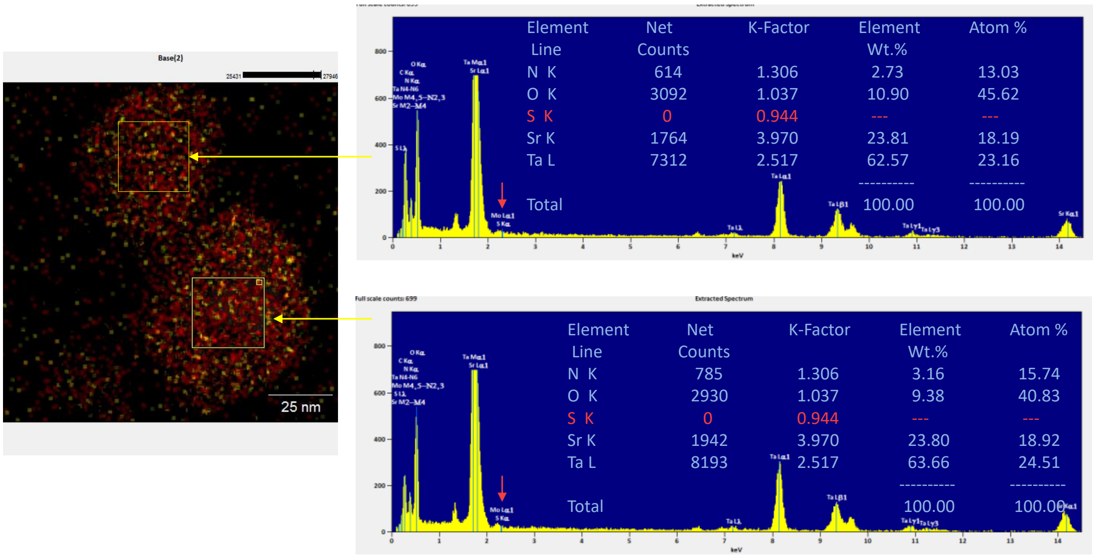

Supplementary Fig. 4. STEM–EDS analysis of selected particles in the cross-sectional sample of $SrTaO_2N$.

Note: This EDS result indicates that sulfur was not incorporated into the $SrTaO_2N$ framework, consistent with the elemental analysis results (Supplementary Table 1).

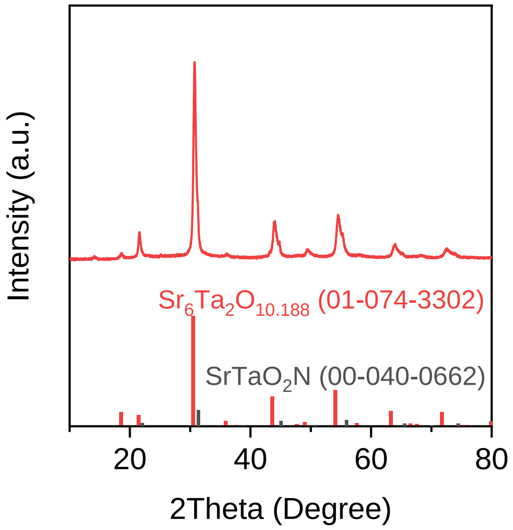

Supplementary Fig. 5. XRD pattern for the product generated by nitridation of the Ta_2O_5/$Sr(OH)_2$/$SrCl_2$ (molar ratio: 0.5/2.5/1) mixture under a NH_3 flow of 200 mL min^{-1} at 1223 K for 3 h.

Note: Nitridation of the Ta_2O_5/$Sr(OH)_2$/$SrCl_2$ (molar ratio: 0.5/2.5/1) mixture did not produce $SrTaO_2N$ but generated predominantly $Sr_6Ta_2O_{10.188}$. This result indicates the indispensable role of TaS_2 as the Ta source in the developed approach.

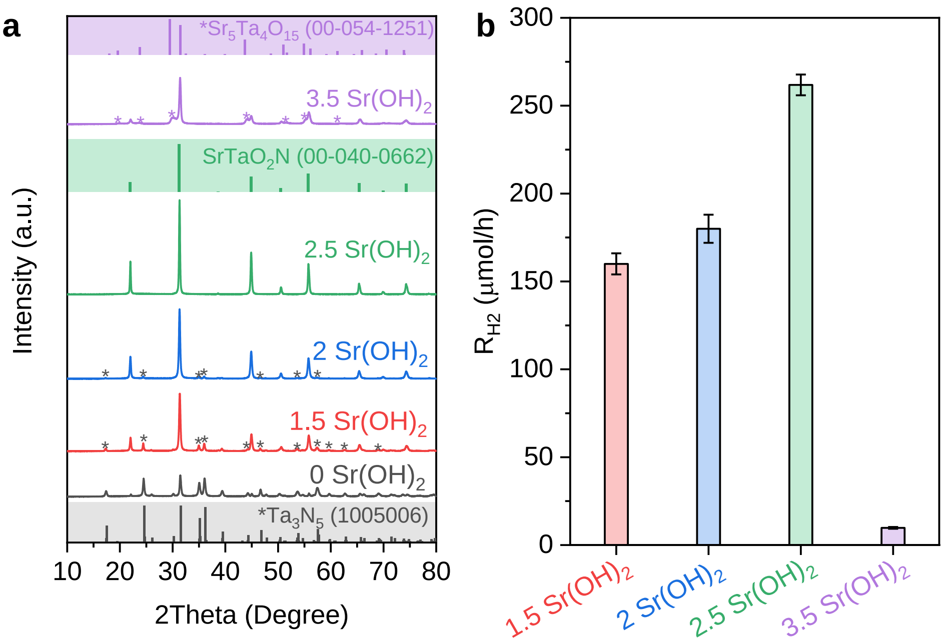

Supplementary Fig. 6. (a) XRD patterns for materials generated from nitridation of mixtures of TaS_2/$Sr(OH)_2$/$SrCl_2$ with molar ratios of 1:*x*:1 (*x* = 0, 1.5, 2, 2.5, 3.5). (b) Photocatalytic H_2-evolution rates for the resulting materials modified with the Cr_2O_3/Pt (MW_{EG})/Ir (MW_{H2O}) (Cr, 0.5 wt%; Pt, 1 wt%; Ir, 0.5 wt%) cocatalysts in an aqueous methanol solution under visible light ($\lambda \geq 420$ nm).

Note: Ta_3N_5 with poor crystallinity was formed when $Sr(OH)_2$ was absent from the precursor, as reflected by the low XRD intensity for Ta_3N_5 (grey spectrum in Supplementary Fig. 6a). The increase in the proportion of $Sr(OH)_2$ inhibited the formation of Ta_3N_5 while promoting the formation of $SrTaO_2N$, and single-phase $SrTaO_2N$ was obtained when the TaS_2/$Sr(OH)_2$/$SrCl_2$ molar ratio was 1/2.5/1. Excess $Sr(OH)_2$ (e.g., a TaS_2/$Sr(OH)_2$/$SrCl_2$ molar ratio of 1/3.5/1) notably suppressed the formation of well-crystallized $SrTaO_2N$ but promoted the formation of $Sr_5Ta_4O_{15}$ as a byproduct.

The photocatalytic H_2-evolution result concerning the effect of the $Sr(OH)_2$ content in the precursor (Supplementary Fig. 6b) indicates that the optimal TaS_2/$Sr(OH)_2$/$SrCl_2$ molar ratio in the precursor is 1/2.5/1.

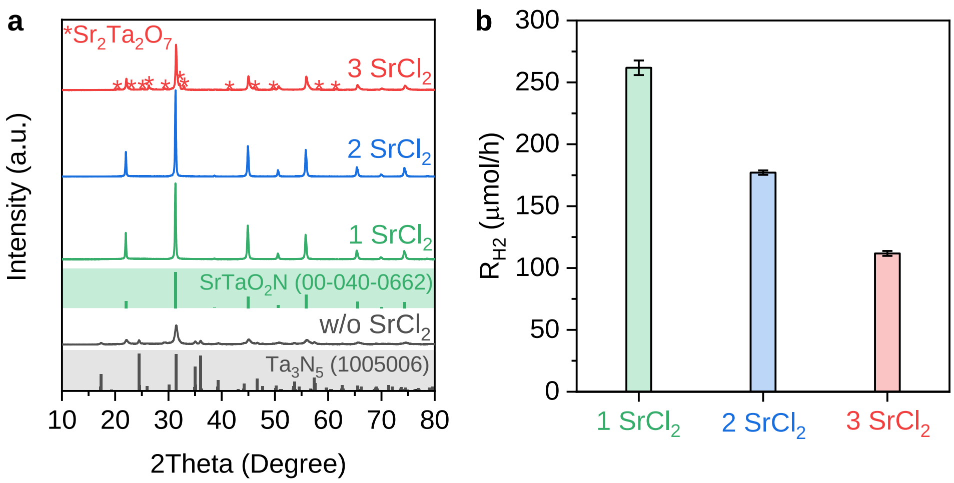

Supplementary Fig. 7. (a) XRD patterns for the materials generated by nitridation of mixtures of $TaS_2/Sr(OH)_2/SrCl_2$ with molar ratios of 1:2.5:y (y = 0, 1, 2, 3). (b) Photocatalytic H_2-evolution rates for the resultant materials modified with the Cr_2O_3/Pt (MW_{EG})/Ir (MW_{H2O}) (Cr, 0.5 wt%; Pt, 1 wt%; Ir, 0.5 wt%) cocatalysts in an aqueous methanol solution under visible light ($\lambda \geq 420$ nm).

Note: The absence of $SrCl_2$ from the precursor resulted in the formation of a mixture of $SrTaO_2N$ and Ta_3N_5 with very poor crystallinity, as reflected by the low XRD intensities for both components (grey spectrum in Supplementary Fig. 7a). Upon addition of $SrCl_2$ with a $TaS_2/Sr(OH)_2/SrCl_2$ molar ratio of 1/2.5/1 or 1/2.5/2, single-phase $SrTaO_2N$ was obtained. Nevertheless, an excess amount of $SrCl_2$ in the precursor (e.g., a $TaS_2/Sr(OH)_2/SrCl_2$ molar ratio of 1/2.5/3) resulted in the formation of $Sr_2Ta_2O_7$ as a byproduct.

The photocatalytic H_2-evolution result related to the effect of the $SrCl_2$ content in the precursor (Supplementary Fig. 7b) indicates that the optimal $TaS_2/Sr(OH)_2/SrCl_2$ molar ratio in the precursor is 1/2.5/1.

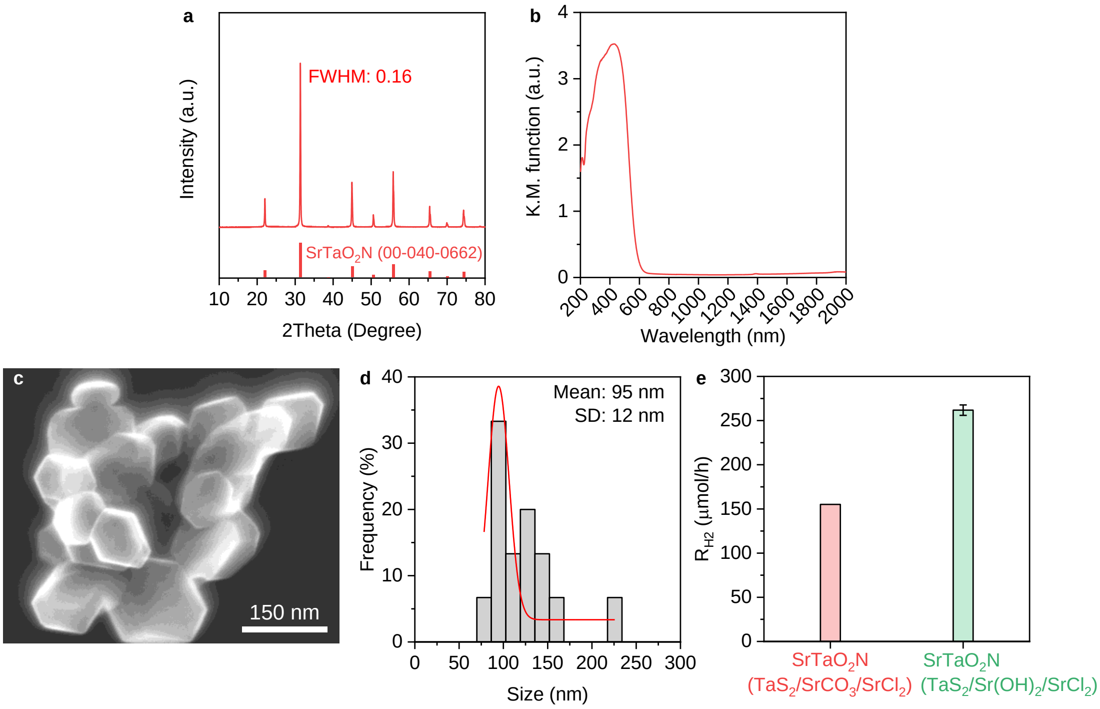

Supplementary Fig. 8. (a) XRD pattern, (b) diffuse-reflectance spectrum, (c) SEM image, and (d) particle size distribution for the material generated by nitridation of mixtures of $TaS_2/SrCO_3/SrCl_2$ with molar ratios of 1:2.5:1. The mean value and standard deviation (SD) of the particle sizes in subfigure d were determined by Gaussian fitting (red line). (e) Comparison of the photocatalytic H_2-evolution rates for the resultant material (i.e., $SrTaO_2N$ ($TaS_2/SrCO_3/SrCl_2$)) and $SrTaO_2N$ ($TaS_2/Sr(OH)_2/SrCl_2$) modified with the Cr_2O_3/Pt (MW_{EG})/Ir (MW_{H2O}) (Cr, 0.5 wt%; Pt, 1 wt%; Ir, 0.5 wt%) cocatalysts in an aqueous methanol solution under visible light ($\lambda \geq 420$ nm).

Note: This result indicates that $Sr(OH)_2$ is a superior source of strontium and oxygen compared with $SrCO_3$ in the developed approach, generating $SrTaO_2N$ nanocrystals with smaller particle sizes and greater activity toward photocatalytic H_2 evolution.

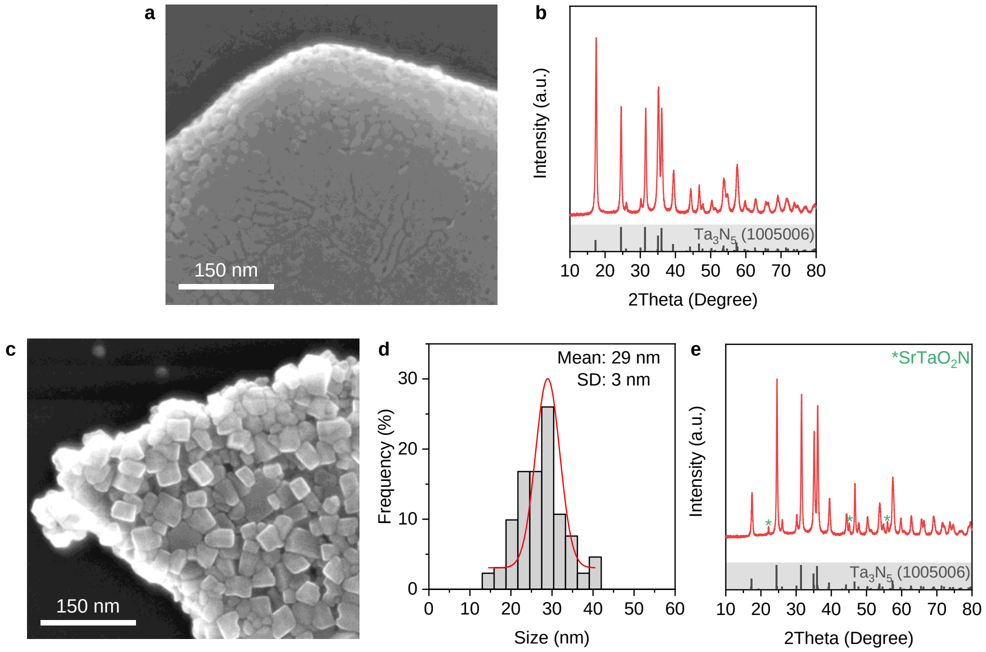

Supplementary Fig. 9. (a) SEM image and (b) XRD pattern of the material generated by nitridation of TaS_2 at 1223 K for 3 h. (c) SEM image, (d) particle-size distribution and (e) XRD pattern of the material generated by nitridation of $TaS_2/SrCl_2$ with a molar ratio of 1:1 at 1223 K for 0.5 h. The mean value and standard deviation (SD) of the particle sizes in subfigure d were determined by Gaussian fitting (red line).

Note: As shown in Supplementary Fig. 9a and b, direct nitridation of TaS_2 under NH_3 generated Ta_3N_5 retaining the several-micron-sized stacked-layer structure of TaS_2 (Supplementary Fig. 1a). In contrast, nitridation of TaS_2 in the presence of $SrCl_2$ for 0.5 h formed aggregates dominantly composed of monodispersed Ta_3N_5 nanoparticles with an average size of 29 nm (Supplementary Fig. 9c-e). The generation of minor $SrTaO_2N$ in the latter case is due to the favorable water uptake by $SrCl_2$ in the precursor. This difference indicates that the $SrCl_2$ molten-salt could promote the decomposition of TaS_2 into nanoscale fragments during the thermal nitridation process, which is presumably a key reason for the formation of *A*TaO_2N single nanocrystals upon thermal nitridation of a mixture of $TaS_2/Sr(OH)_2/SrCl_2$.

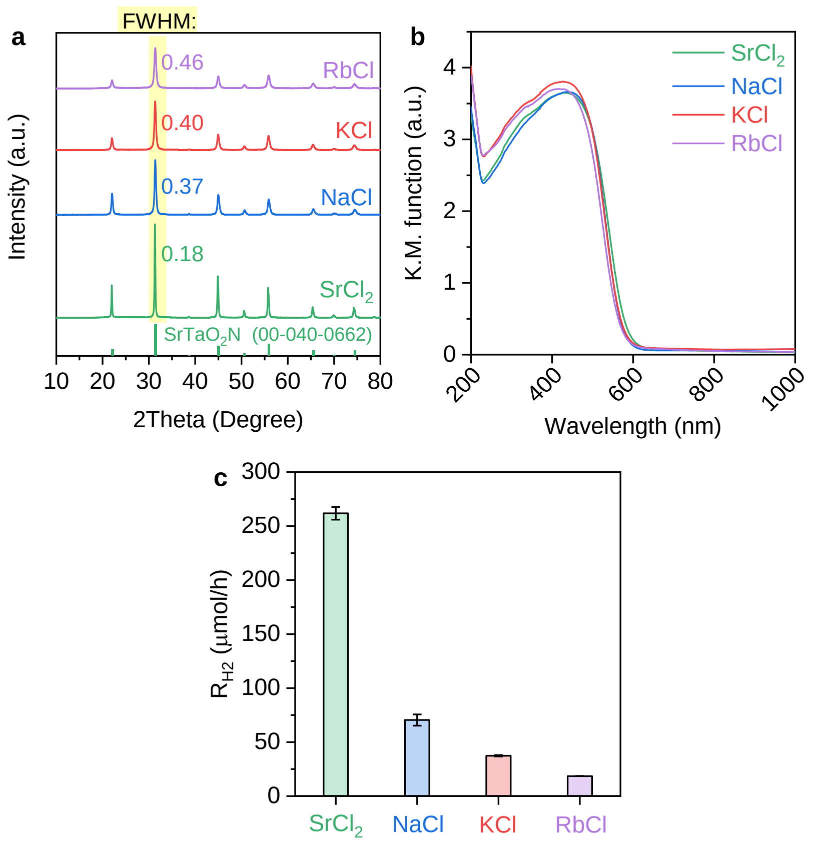

Supplementary Fig. 10. (a) XRD patterns, (b) UV–vis diffuse-reflectance spectra, and (c) photocatalytic H_2-evolution rates for different $SrTaO_2N$ nanocrystals prepared from different molten-salt fluxes. The precursors for nitridation are mixtures of $TaS_2/Sr(OH)_2/SrCl_2$, $TaS_2/Sr(OH)_2/NaCl$, $TaS_2/Sr(OH)_2/KCl$, and $TaS_2/Sr(OH)_2/RbCl$ with the same molar ratio of 1:2.5:1. The same Cr_2O_3/Pt (MW_{EG})/Ir (MW_{H2O}) (Cr, 0.5 wt%; Pt, 1 wt%; Ir, 0.5 wt%) cocatalysts were used to decorate these different $SrTaO_2N$ samples for H_2 evolution.

Note: As reflected by the full-width at half-maximum (FWHM) values of the characteristic XRD peak at 31.4°, the degree of crystallinity of the resultant $SrTaO_2N$ follows the sequence $SrTaO_2N$ ($TaS_2/Sr(OH)_2/SrCl_2$) > $SrTaO_2N$ ($TaS_2/Sr(OH)_2/NaCl$) > $SrTaO_2N$ ($TaS_2/Sr(OH)_2/KCl$) > $SrTaO_2N$ ($TaS_2/Sr(OH)_2/RbCl$). This result appears to be related to the melting point of the molten salt utilized, which is 1147, 1074, 1043, and 991 K for $SrCl_2$, NaCl, KCl, and RbCl, respectively. The higher the melting point, the greater the

crystallinity of the resultant $SrTaO_2N$. Accordingly, $SrTaO_2N$ nanocrystals prepared with $SrCl_2$ outperformed those prepared with other fluxes. This result indicates that the developed approach enables the use of different molten-salt fluxes to fabricate single-crystal $SrTaO_2N$ with tunable crystallinities and photocatalytic activities.

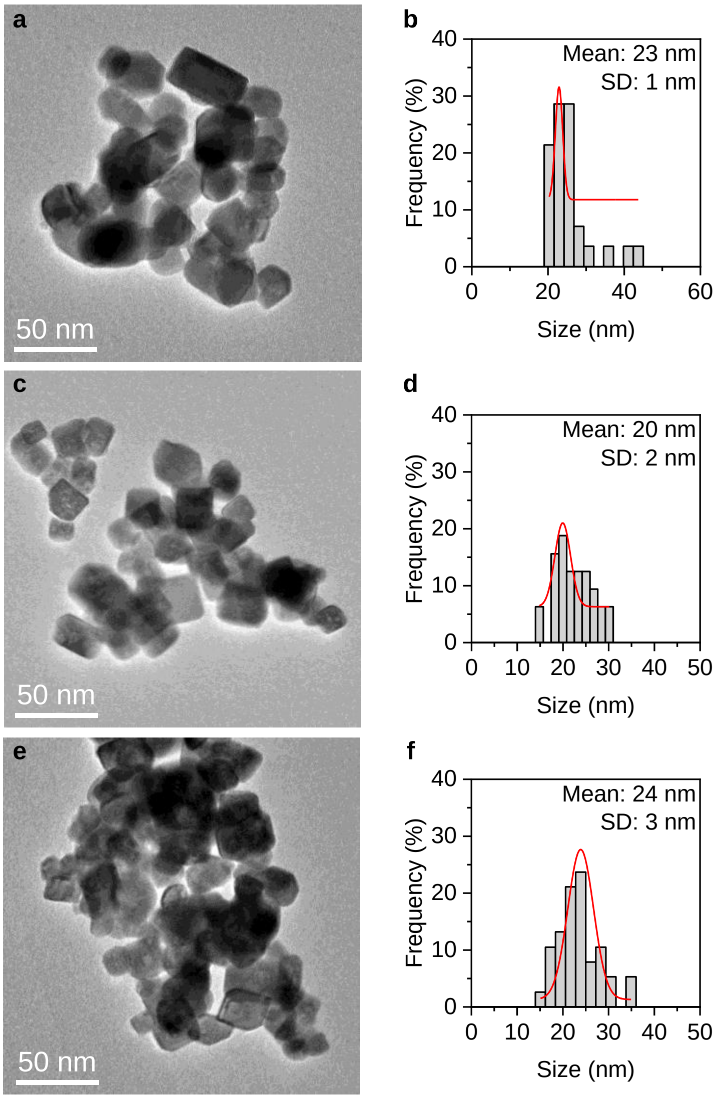

Supplementary Fig. 11. (a, c, and e) Bright-field TEM images and (b, d, and f) particle size distributions for the $SrTaO_2N$ prepared with NaCl (a and b), KCl (c and d), and RbCl (e and f) molten-salt fluxes. The mean value and standard deviation (SD) of the particle sizes in b, d, and f were determined by Gaussian fitting (red lines).

Note: The average particle size for the $SrTaO_2N$ prepared by nitridation of mixtures of $TaS_2/Sr(OH)_2/NaCl$, $TaS_2/Sr(OH)_2/KCl$, and $TaS_2/Sr(OH)_2/RbCl$ with a molar ratio of 1:2.5:1 was approximately 20 nm.

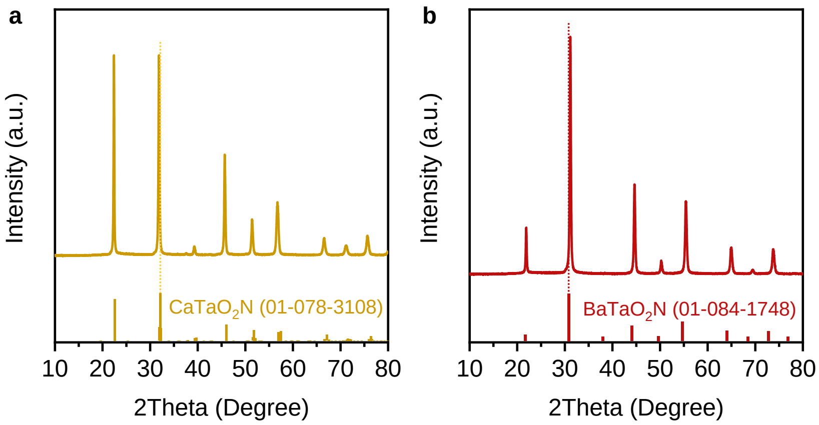

Supplementary Fig. 12. XRD patterns for (a) $CaTaO_2N$ and (b) $BaTaO_2N$ generated by nitridation of mixtures of TaS_2/$Ca(OH)_2$/$SrCl_2$ and TaS_2/$Ba(OH)_2$/$SrCl_2$, respectively, with a molar ratio of 1:2.5:1.

Note: Slight peak shifts to smaller and larger angles were observed in the XRD patterns for the developed $CaTaO_2N$ and $BaTaO_2N$ nanocrystals, respectively. These shifts are attributed to the substitution of *A* sites (Ca^{2+} in $CaTaO_2N$ and Ba^{2+} in $BaTaO_2N$) in both samples by trace Sr^{2+} originating from the $SrCl_2$ flux in the precursor. This observation is consistent with the red and blue shifts observed in the UV–vis diffuse-reflectance spectra of the developed $CaTaO_2N$ and $BaTaO_2N$ (Fig. 1j), respectively, compared with the typical peak positions in the spectra of their counterparts.

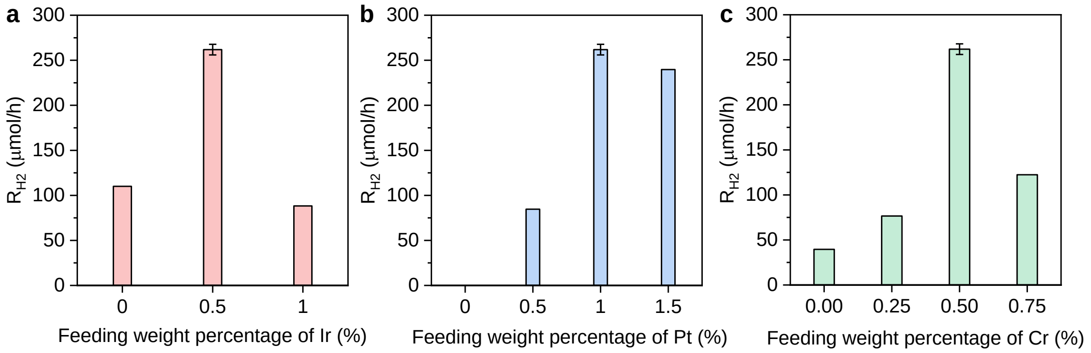

Supplementary Fig. 13. (a) Photocatalytic H_2-evolution rates over $SrTaO_2N$ nanocrystals as a function of the fed Ir concentration (relative to the mass of $SrTaO_2N$) in the Cr_2O_3/Pt (MW_{EG})/Ir (MW_{H2O}) cocatalyst (0.5 wt% Cr/1.0 wt% Pt/*x* wt% Ir with varying *x*). (b) Photocatalytic H_2-evolution rates over $SrTaO_2N$ nanocrystals as a function of the fed Pt concentration (relative to the mass of $SrTaO_2N$) in the Cr_2O_3/Pt (MW_{EG})/Ir (MW_{H2O}) cocatalyst (*z* wt% Cr/*y* wt% Pt/0.5 wt% Ir (*y*/*z* = 2) with varying *y*). (c) Photocatalytic H_2-evolution rates over $SrTaO_2N$ nanocrystals as a function of the fed Cr concentration (relative to the mass of $SrTaO_2N$) in the Cr_2O_3/Pt (MW_{EG})/Ir (MW_{H2O}) cocatalyst (*z* wt% Cr/1 wt% Pt/0.5 wt% Ir with varying *z*).

Note: These results indicate that the optimized feeding concentrations of Ir, Pt, and Cr (relative to the mass of $SrTaO_2N$) in the Cr_2O_3/Pt (MW_{EG})/Ir (MW_{H2O}) cocatalyst were 0.5, 1.0, and 0.5 wt%, respectively; these concentrations led to the highest photocatalytic H_2-evolution rate.

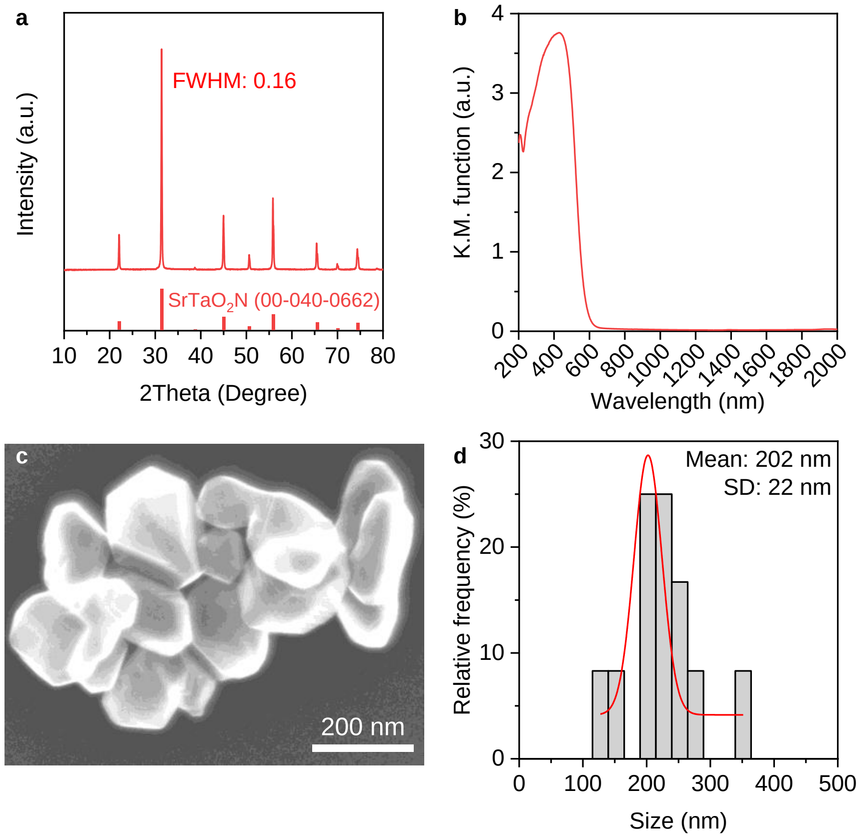

Supplementary Fig. 14. (a) XRD pattern, (b) diffuse-reflectance spectrum, (c) SEM image, and (d) particle size distribution for the material generated by nitridation of a mixture of $Ta_2O_5/SrCO_3/SrCl_2$ with a molar ratio of 0.5:1:1. The mean value and standard deviation (SD) of the particle sizes in subfigure d were determined by Gaussian fitting (red line).

Note: This sample is a reference $SrTaO_2N$ sample prepared by nitridation of typical oxide and carbonate precursors together with a molten-salt flux. Note that a $Ta_2O_5/SrCO_3/SrCl_2$ molar ratio of 0.5:1:1 was used because this ratio was optimized to obtain single-phase $SrTaO_2N$. Note also that single-phase $SrTaO_2N$ could not be formed when the $Ta_2O_5/SrCO_3/SrCl_2$ molar ratio was 0.5:2.5:1. The crystallinity of this $SrTaO_2N$ sample was similar to that of the $SrTaO_2N$ nanocrystals synthesized from $TaS_2/Sr(OH)_2/SrCl_2$ with a molar ratio of 1:2.5:1, as reflected by the similar FWHM values for their characteristic XRD peak at 31.4°. However, the average particle size was approximately four times larger.

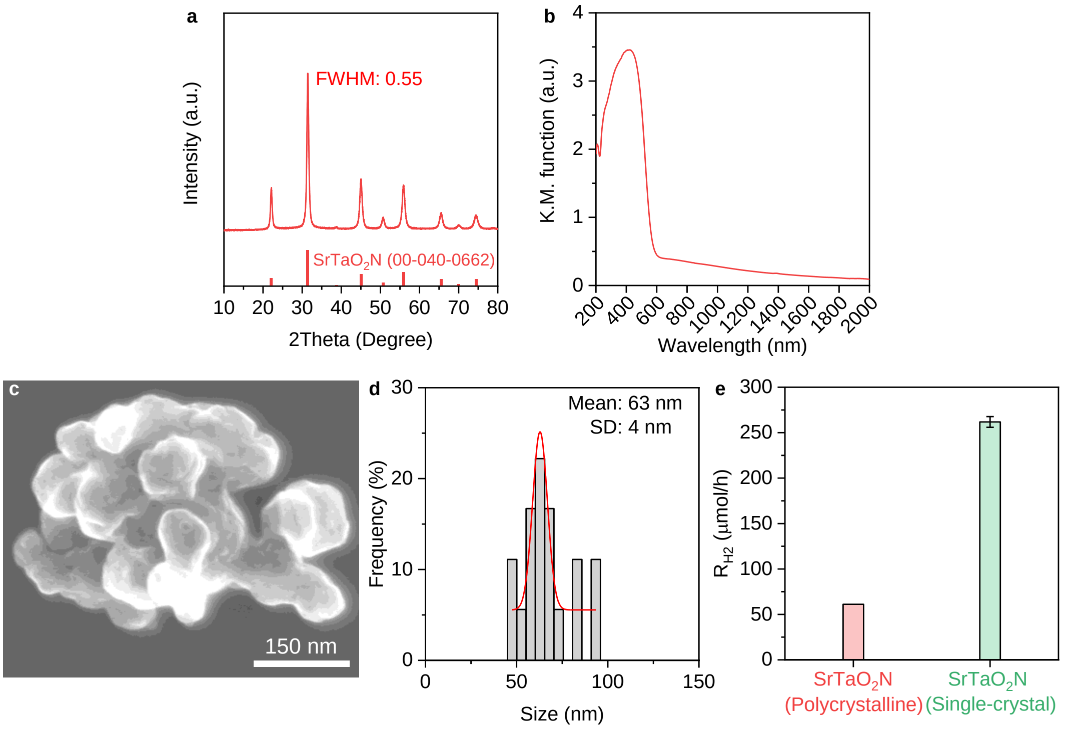

Supplementary Fig. 15. (a) XRD pattern, (b) diffuse-reflectance spectrum, (c) SEM image, and (d) particle size distribution for the synthesized polycrystalline $SrTaO_2N$. The mean value and standard deviation (SD) of the particle sizes in subfigure d were determined by Gaussian fitting (red line). (e) Comparison of the photocatalytic H_2-evolution rates for the polycrystalline $SrTaO_2N$ and single-nanocrystal $SrTaO_2N$ modified with the same Cr_2O_3/Pt (MW_{EG})/Ir (MW_{H2O}) (Cr, 0.5 wt%; Pt, 1 wt%; Ir, 0.5 wt%) cocatalysts in an aqueous methanol solution under visible light ($\lambda \geq 420$ nm).

Note: This sample is a reference $SrTaO_2N$ sample prepared by calcination of the single-nanocrystal $SrTaO_2N$ (Fig. 1a-i) in air at 1173 K for 1 h (yielding $Sr_2Ta_2O_7$) followed with nitridation of the resultant material in flowing NH_3 (200 mL/min) at 1223 K for 3 h. The synthesized polycrystalline $SrTaO_2N$ exhibits aggregates composed of several polycrystalline nanoparticles with an average size of 63 nm, which is close to that of single-nanocrystal $SrTaO_2N$ (52 nm). However, the crystallinity of this polycrystalline $SrTaO_2N$ sample (FWHM: 0.55 degrees) was significantly worse than that of the single-nanocrystal $SrTaO_2N$ (FWHM: 0.18 degrees), as indicated by the largely increased FWHM value for

the characteristic XRD peak at 31.4°. As a result, the photocatalytic H_2 evolution rate was only 66 μmol/h, 4.3 times lower than that of the single-nanocrystal $SrTaO_2N$. This result demonstrates the equal importance of a high degree of crystallinity in addition to a small particle size and the superiority of the developed approach in producing highly crystalline single nanocrystals of *A*TaO_2N (*A* = Sr, Ca, Ba).

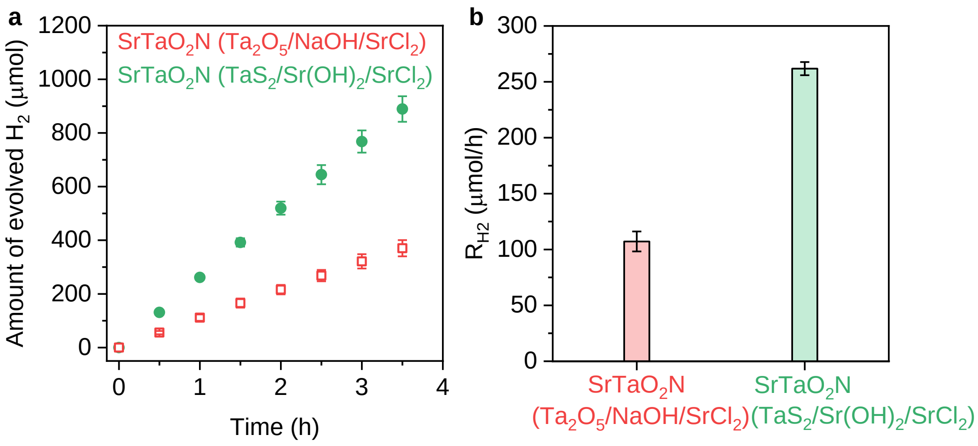

Supplementary Fig. 16. (a) Time courses for photocatalytic H_2 evolution and (b) the associated H_2-evolution rates over the previously developed $SrTaO_2N$ (prepared from a Ta_2O_5/NaOH/$SrCl_2$ precursor[1]) and single-nanocrystal $SrTaO_2N$ (prepared from a TaS_2/$Sr(OH)_2$/$SrCl_2$ precursor) modified with the same Cr_2O_3/Pt (MW_{EG})/Ir (MW_{H2O}) (Cr, 0.5 wt%; Pt, 1 wt%; Ir, 0.5 wt%) cocatalysts in an aqueous methanol solution under visible light ($\lambda \geq 420$ nm).

Note: The reference $SrTaO_2N$ (Ta_2O_5/NaOH/$SrCl_2$) specimen was prepared according to our previously reported method (i.e., thermal nitridation of a Ta_2O_5/NaOH/$SrCl_2$ precursor with a molar ratio of 1:1:4 under 200 mL/min NH_3 at 1223 K for 5 h)[1], which contained a pinch of Ta_3N_5 as a byproduct and exhibited a relatively wide particle-size distribution (70–250 nm). This reference sample (R_{H2} = 107 μmol/h) exhibited 2.4 times lower performance for photocatalytic H_2 evolution compared with the single-nanocrystal $SrTaO_2N$ (R_{H2} = 262 μmol/h) developed in this work.

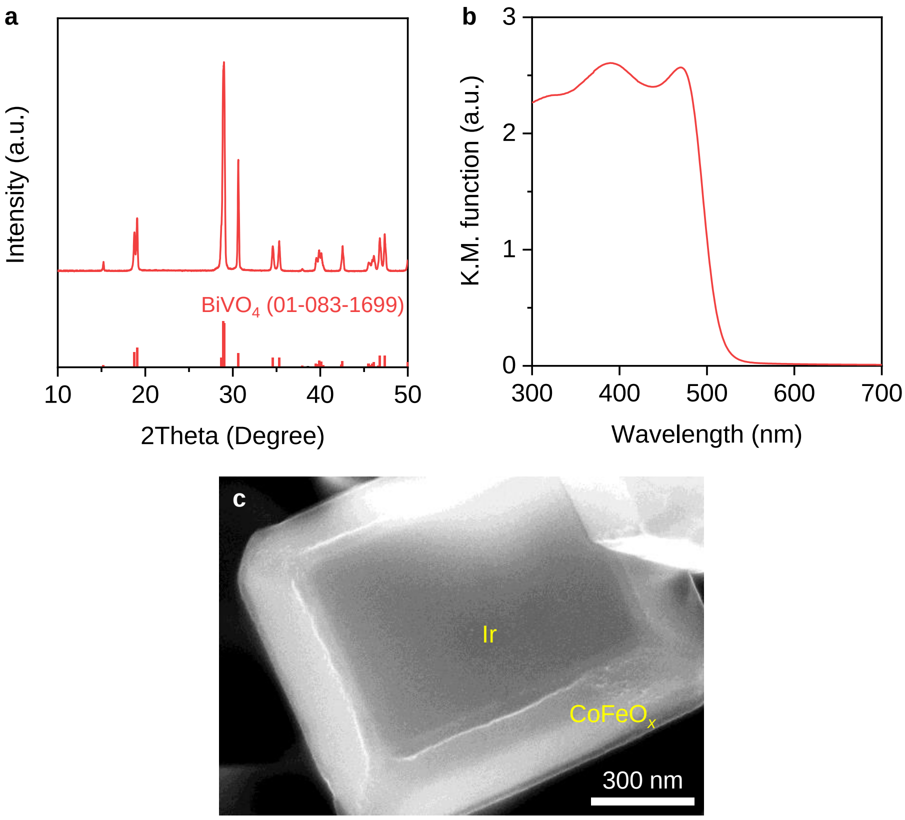

Supplementary Fig. 17. (a) XRD pattern and (b) UV–vis diffuse-reflectance spectrum for $BiVO_4$. (c) SEM image of the Ir-FeCoO$_x$/$BiVO_4$.

Note: This Ir-FeCoO$_x$/$BiVO_4$ material was prepared according to a recently reported method[2], and similar characterization results were obtained herein.

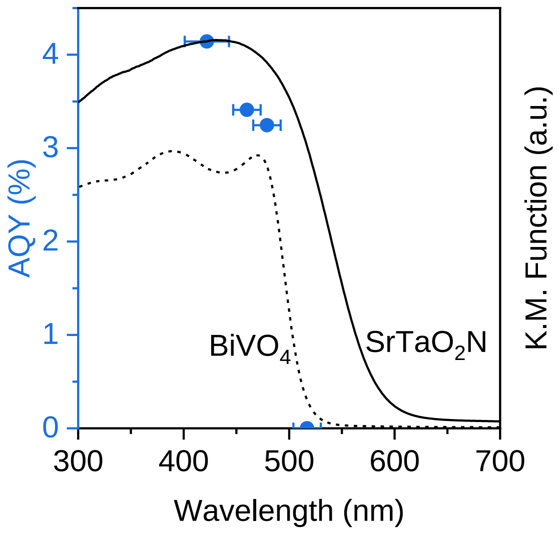

Supplementary Fig. 18. AQY as a function of the incident-light wavelength during Z-scheme OWS using Cr_2O_3/Pt (MW_{EG})/Ir (MW_{H2O})-modified $SrTaO_2N$ nanocrystals as the HEP, Ir-$CoFeO_x$/$BiVO_4$ as the OEP, and $[Fe(CN)_6]^{3-}$/$[Fe(CN)_6]^{4-}$ as a redox mediator. Conditions: Cr_2O_3/Pt (MW_{EG})/Ir (MW_{H2O})/$SrTaO_2N$, 50 mg; Ir-$CoFeO_x$/$BiVO_4$, 100 mg; 150 mL of 5 mM $K_4[Fe(CN)_6]$ solution; light source, a Xe lamp (300 W) with various bandpass filters; background pressure, 5 kPa. The solid and dashed lines indicate UV–vis diffuse-reflectance spectra of $SrTaO_2N$ and $BiVO_4$, respectively.

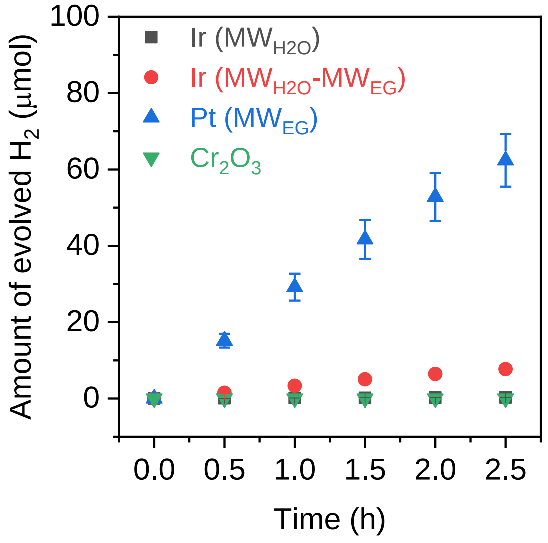

Supplementary Fig. 19. Time course of photocatalytic H_2 evolution over $SrTaO_2N$ nanocrystals modified with different single-component cocatalysts. Note that Ir (MW_{H2O}-MW_{EG})-modified $SrTaO_2N$ indicates the sample obtained after Ir (MW_{H2O})/$SrTaO_2N$ was subjected to a similar microwave treatment in EG.

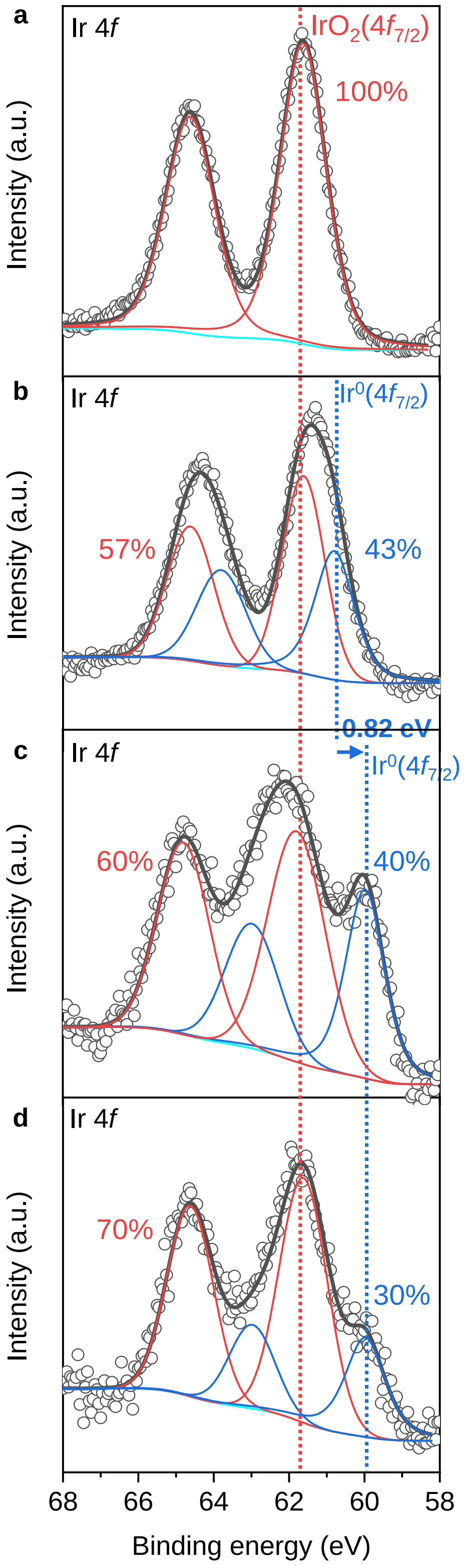

Supplementary Fig. 20. Ir 4*f* XPS spectra of (a) Ir (MW_{H2O})-, (b) Ir (MW_{H2O}-MW_{EG})-, (c) Pt (MW_{EG})/Ir (MW_{H2O})-, and (d) Cr_2O_3/Pt (MW_{EG})/Ir (MW_{H2O})-modified $SrTaO_2N$ nanocrystals. The percentages shown in this figure indicate the surface contents of different Ir species.

Note: The Ir 4*f* peak could be fitted using two doublets (4$f_{7/2}$–4$f_{5/2}$) with a spin–orbit splitting of 3.0 eV and a fixed area ratio of 4:3. Ir (MW_{H2O})-modified $SrTaO_2N$ exhibited a single Ir 4$f_{7/2}$ component with a binding energy of 61.6 eV, corresponding to IrO_2[3]. The binding energies of the Ir 4$f_{7/2}$ components in Ir (MW_{H2O}-MW_{EG})-modified $SrTaO_2N$ were 61.6 and 60.8 eV, which were typically assigned to IrO_2 and Ir^0, respectively[3]. This result indicates that, when Ir (MW_{H2O})/$SrTaO_2N$ was subjected to an EG-mediated microwave treatment (forming Ir (MW_{H2O}-MW_{EG})/$SrTaO_2N$), approximately 43% of the IrO_2 species was reduced to metallic Ir.

A notable shoulder peak emerged at approximately 60.0 eV (Supplementary Fig. 20c and d), constituting the Ir 4$f_{7/2}$ component assigned to Ir^0, in the spectra of the Pt (MW_{EG})/Ir (MW_{H2O})- and Cr_2O_3/Pt (MW_{EG})/Ir (MW_{H2O})-modified $SrTaO_2N$. This shoulder peak indicates the formation of Ir^0, consistent with the Ir^0 formed in Ir (MW_{H2O}-MW_{EG})-modified $SrTaO_2N$; however, the binding energy of Ir^0 in the spectra of Pt (MW_{EG})/Ir (MW_{H2O})- and Cr_2O_3/Pt (MW_{EG})/Ir (MW_{H2O})-modified $SrTaO_2N$ is shifted ~0.8 eV lower than that in the spectrum of Ir (MW_{H2O}-MW_{EG})-modified $SrTaO_2N$. This unique change is most likely due to the interaction between Ir^0 and Pt^0.

In addition, the Ir 4*f* XPS spectra of Pt (MW_{EG})/Ir (MW_{H2O})- and Cr_2O_3/Pt (MW_{EG})/Ir (MW_{H2O})-modified $SrTaO_2N$ are approximately the same, indicating that photodeposition of Cr_2O_3 did not alter the chemical state of the Ir species.

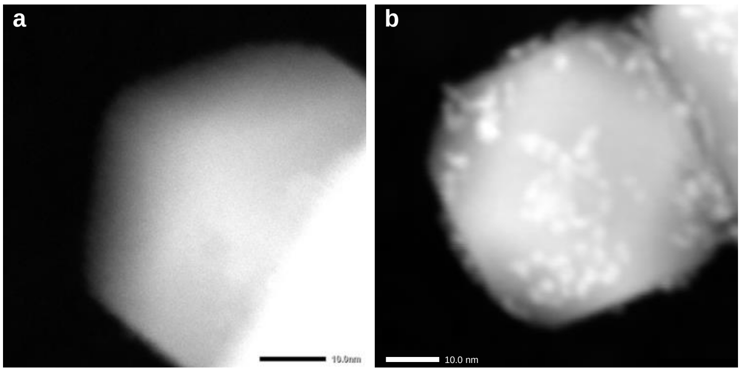

Supplementary Fig. 21. Dark-field STEM image of (a) Ir (MW_{H2O})- and (b) Pt (MW_{EG})/Ir (MW_{H2O})-modified $SrTaO_2N$ nanocrystals.

Note: Ir species in the Ir (MW_{H2O})-modified $SrTaO_2N$ nanocrystals (Supplementary Fig. 21a) were not distinguishable by STEM, indicating that they are highly dispersed.

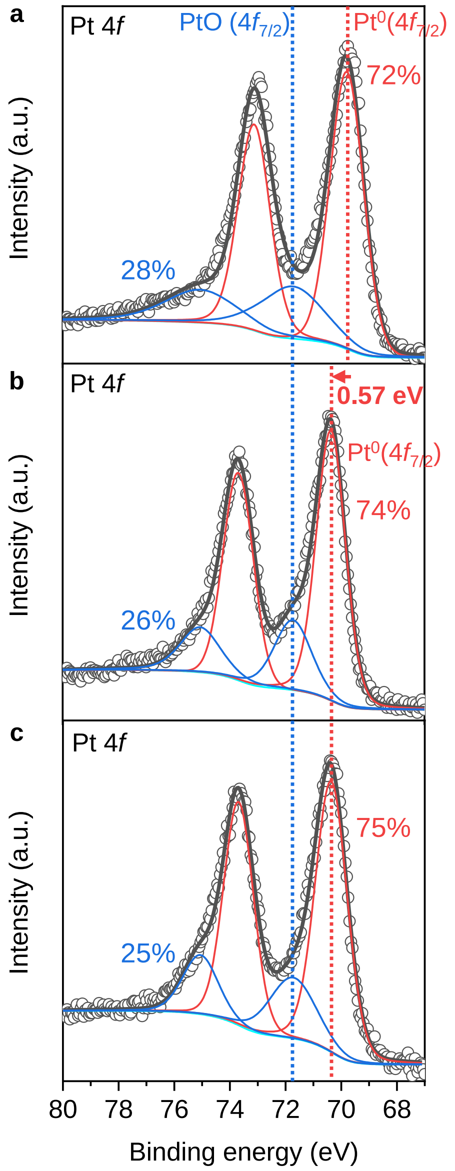

Supplementary Fig. 22. Pt 4*f* XPS spectra of (a) Pt (MW_{EG})-, (b) Pt (MW_{EG})/Ir (MW_{H2O})-, and (d) Cr_2O_3/Pt (MW_{EG})/Ir (MW_{H2O})-modified $SrTaO_2N$ nanocrystals. The percentages shown in this figure indicate the surface contents of different Pt species.

Note: The Pt 4*f* peaks could be fitted using two doublets (Pt $4f_{7/2}$–Pt $4f_{5/2}$) with a spin–orbit splitting of 3.33 eV and a fixed area ratio of 4:3. The binding energies of the Ta $4f_{7/2}$ components in the spectra of the Pt (MW_{EG})-modified $SrTaO_2N$ were approximately 69.8 and 71.7 eV, corresponding to metallic Pt (dominant) and PtO, respectively[4,5]. However, the Pt 4*f* binding energy for Pt^0 in the spectra of Pt (MW_{EG})/Ir (MW_{H2O})- and Cr_2O_3/Pt (MW_{EG})/Ir

(MW_{H2O})-modified $SrTaO_2N$ was positively shifted compared with that in the spectrum of Pt (MW_{EG})-modified $SrTaO_2N$ by approximately 0.6 eV (to 70.4 eV). This unique change is most likely due to the interaction between Ir^0 and Pt^0, consistent with the negative binding-energy shift observed for Ir^0 (Supplementary Fig. 20c and d).

The Pt 4*f* XPS spectra of Pt (MW_{EG})/Ir (MW_{H2O})- and Cr_2O_3/Pt (MW_{EG})/Ir (MW_{H2O})-modified $SrTaO_2N$ are approximately the same, indicating that photodeposition of Cr_2O_3 did not alter the chemical state of the Pt species.

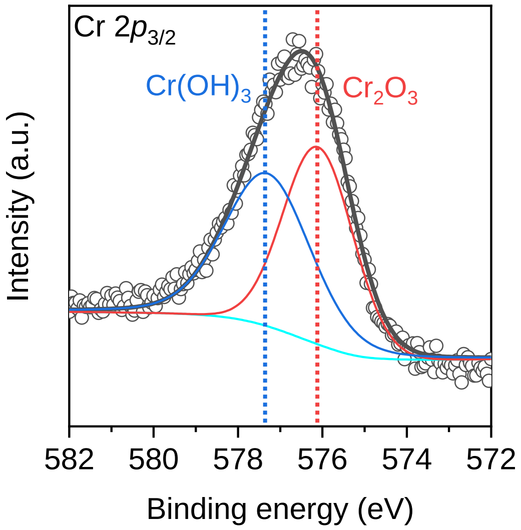

Supplementary Fig. 23. Cr 2$p_{3/2}$ XPS spectrum of Cr_2O_3/Pt (MW_{EG})/Ir (MW_{H2O})-modified $SrTaO_2N$ nanocrystals.

Note: Cr_2O_3 and $Cr(OH)_3$ species with binding energies of 576.1 (this peak could be further fitted using a multiplet) and 577.3 eV, respectively, were detected by XPS, consistent with a previous study in which a photodeposited Cr shell was composed of Cr(III)$O_{1.5-m}(OH)_{2m}\cdot xH_2O$[5]. However, it is labeled as Cr_2O_3 herein for the sake of simplicity.

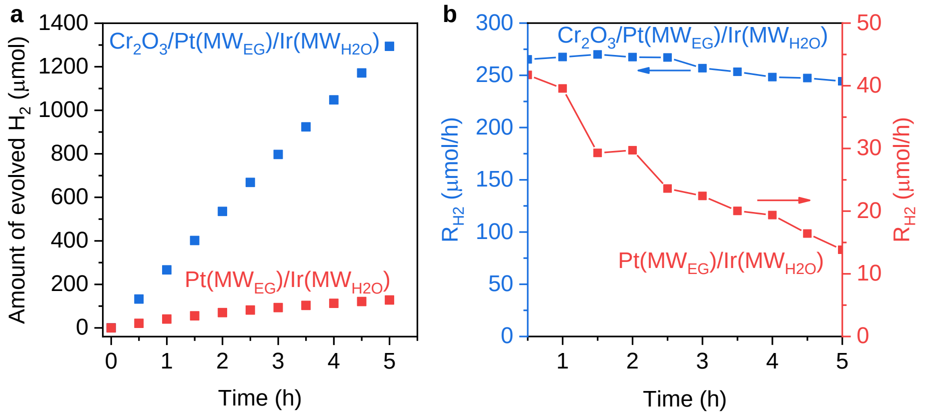

Supplementary Fig. 24. (a) Typical time courses of photocatalytic H_2 evolution over Cr_2O_3/Pt (MW_{EG})/Ir (MW_{H2O})- and Pt (MW_{EG})/Ir (MW_{H2O})-modified $SrTaO_2N$ nanocrystals. (b) Instant photocatalytic H_2-evolution rates (calculated every 30 min) for Cr_2O_3/Pt (MW_{EG})/Ir (MW_{H2O})- and Pt (MW_{EG})/Ir (MW_{H2O})-modified $SrTaO_2N$ nanocrystals as a function of the reaction time.

Note: This result indicates that the deposition of Cr_2O_3 as an overlayer covering the Ir–Pt alloy nanoparticle not only improved the H_2-evolution kinetics (Supplementary Fig. 24a) but also notably improved the photocatalytic durability (Supplementary Fig. 24b) because the oxidized species generated by holes were prevented from reaching the metal core and being reduced back.

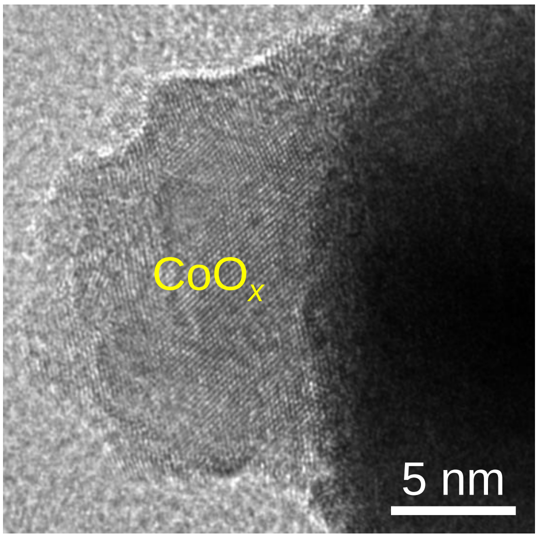

Supplementary Fig. 25. Bright-field TEM image of the CoO_x cocatalyst on the surface of a $SrTaO_2N$ nanocrystal.

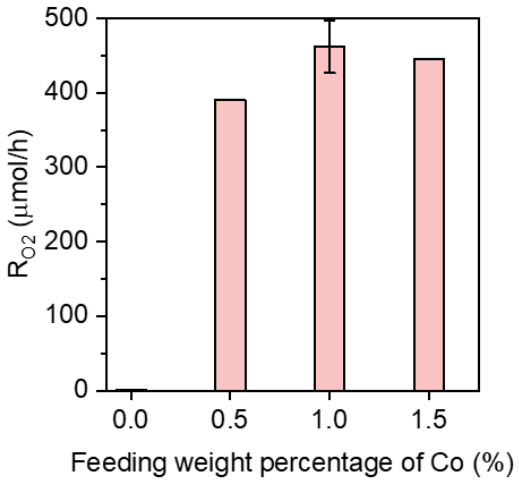

Supplementary Fig. 26. Photocatalytic O_2 evolution rates for $SrTaO_2N$ nanocrystals as a function of the concentration of deposited CoO_x cocatalyst (relative to the mass of $SrTaO_2N$).

Supplementary Table 1: Chemical composition of the prepared $SrTaO_2N$.

Element	Detected amount (mmol/g)	Estimated chemical formula
Sr[a]	3.04804	$Sr_{1.00}Ta_{1.01}O_{2.07}N_{1.03}$
Ta[a]	3.07766	
O[b]	6.32034	
N[b]	3.13862	
S[b]	0.00123[c]	

[a]Determined by the inductively coupled plasma-atomic emission spectroscopy (ICP-AES; ICPS-8100, Shimadzu). [b]Determined by an oxygen-nitrogen-sulfur combustion analyzer (Horiba, EMGA-620W).[c]This value is equal to 0.004 wt%, below the detection limit of the instrument (0.01 wt%).

Supplementary Table 2: Comparison of photocatalytic H_2/O_2 evolution performance between representative $SrTaO_2N$ photocatalysts in the literature and the $SrTaO_2N$ nanocrystal photocatalysts in this work.

Photocatalyst material (Morphology & size)	Precursor for nitridation	Cocatalyst modification	Reaction solution	Gas evolution rates[a] (μmol/h)	AQY
$SrTaO_2N$ (sub-50 nm single nanocrystals, this work)	$TaS_2/Sr(OH)_2/SrCl_2$	$Ir\text{-}Pt/Cr_2O_3$	Methanol solution	H_2: ca. 260 μmol/h	3% at 420 nm
Sr-doped $SrTaO_2N$ (Several-micron-sized particles with pinholes)[6]	$Sr_4Ta_2O_9$	Pt	50 mM Na_2SO_3 solution	H_2: ca. 5 μmol/h	NR
$SrTaO_2N\text{-}CaTaO_2N$ solid solutions (Aggregated microstructure with abundant pores)[7]	polymetallic oxides	Pt	Methanol solution	H_2: ca. 4 μmol/h	NR
Ga-doped $SrTaO_2N$ (50–200 nm particles)[8]	$Ta_2O_5/SrO/SrCl_2$	Pt	Methanol solution	H_2: ca. 2 μmol/h	NR
$SrTaO_2N$ (sub-50 nm single nanocrystals, this work)	$TaS_2/Sr(OH)_2/SrCl_2$	CoO_x	20 mM $AgNO_3$ solution	O_2: ca. 460 μmol/h	9% at 420 nm
$SrTaO_2N$ (Several-micron-sized mesoporous single crystals)[9]	$Sr_2Bi_3TaO_{11}Cl$	CoO_x	50 mM $AgNO_3$ solution	O_2: ca. 100 μmol/h	17.9% at 420 nm
Ga-doped $SrTaO_2N$ (50–200 nm particles)[8]	$Ta_2O_5/SrO/SrCl_2$	CoO_x	50 mM $AgNO_3$ solution	O_2: 22 μmol/h	3.6% at 420 nm
Sr-doped $SrTaO_2N$ (Several-micron-sized particles with pinholes)[6]	$Sr_4Ta_2O_9$	$Rh@Rh_2O_3$	50 mM $AgNO_3$ solution	O_2: ca. 50 μmol/h	3.4% at 420 nm
$SrTaO_2N\text{-}CaTaO_2N$ solid solutions (Aggregated microstructure with abundant pores)[7]	polymetallic oxides	CoO_x	50 mM $AgNO_3$ solution	O_2: ca. 35 μmol/h	1.67% at 420 nm

[a] Light source: 300 W Xe lamp (λ ≥ 420 nm). AQY: Apparent quantum yield; NR: Not reported.

Supplementary References

1. Chen, K. *et al.* Overall water splitting by a $SrTaO_2N$-based photocatalyst decorated with an Ir-promoted Ru-based cocatalyst. *J. Am. Chem. Soc.* **145**, 3839-3843 (2023).

2. Qi, Y. *et al.* Unraveling of cocatalysts photodeposited selectively on facets of $BiVO_4$ to boost solar water splitting. *Nat. Commun.* **13**, 484 (2022).

3. Freakley, S. J., Ruiz-Esquius, J. & Morgan, D. J. The X-ray photoelectron spectra of Ir, IrO_2 and $IrCl_3$ revisited. *Surf. Interface Anal.* **49**, 794-799 (2017).

4. Lian, J. *et al.* Metal-seed assistant photodeposition of platinum over Ta_3N_5 photocatalyst for promoted solar hydrogen production under visible light. *J. Energy Chem.* **55**, 444-448 (2021).

5. Xiao, J. *et al.* Simultaneously tuning the defects and surface properties of Ta_3N_5 nanoparticles by Mg-Zr codoping for significantly accelerated photocatalytic H_2 evolution. *J. Am. Chem. Soc.* **143**, 10059-10064 (2021).

6. Sun, X. Q., Wu, F. F., Liu, G. & Xu, X. X. Enabling efficient visible light photocatalytic water splitting over $SrTaO_2N$ by incorporating Sr in its B site. *J. Mater. Chem. A* **6**, 20760-20768 (2018).

7. Wang, Y., Wei, S. & Xu, X. $SrTaO_2N$-$CaTaO_2N$ solid solutions as efficient visible light active photocatalysts for water oxidation and reduction. *Appl. Catal. B: Environ.* **263**, 118315 (2020).

8. Obata, K., Higashi, T., Ye, F., Katayama, M. & Takanabe, K. Cation-doped $SrTaO_2N$ prepared through a flux method for visible-light-driven water splitting. *ChemPhotoChem* **7**, e202200293 (2023).

9. Yang, L., Fu, Q., Wang, L., Yu, J. & Xu, X. Liberating photocarriers in mesoporous single-crystalline $SrTaO_2N$ for efficient solar water splitting. *Appl. Catal. B: Environ.* **304**, 120934 (2022).
